# Shorter TCR β-Chains Are Highly Enriched During Thymic Selection and Antigen-Driven Selection

**DOI:** 10.3389/fimmu.2019.00299

**Published:** 2019-02-26

**Authors:** Xianliang Hou, Ping Zeng, Xujun Zhang, Jianing Chen, Yan Liang, Jiezuan Yang, Yida Yang, Xiangdong Liu, Hongyan Diao

**Affiliations:** ^1^State Key Laboratory for Diagnosis and Treatment of Infectious Diseases, Collaborative Innovation Center for Diagnosis and Treatment of Infectious Diseases, The First Affiliated Hospital, College of Medicine, Zhejiang University, Hangzhou, China; ^2^College of Materials and Textile, Zhejiang Sci-Tech University, Hangzhou, China

**Keywords:** T cell receptor, cell subsets, deep sequencing, memory T cell, naive T cell

## Abstract

The adaptive immune system uses several strategies to generate a repertoire of T cell receptors (TCR) with sufficient diversity to recognize the universe of potential pathogens. However, it remains unclear how differences in the T cell receptor (TCR) contribute to heterogeneity in T cell state. In this study, we used polychromatic flow cytometry to isolate highly pure CD4^+^/CD8^+^ naive and memory T cells, and applied deep sequencing to characterize corresponding TCR β-chain (TCRβ) complementary-determining region 3 (CDR3) repertoires. We find that shorter TCRβ CDR3s with fewer insertions were highly enriched during thymic selection. Antigen-experienced T cells (memory T cells) harbor shorter CDR3s vs. naive T cells. Moreover, the public TCRβ CDR3 clonotypes within cell subsets or interindividual tend to have shorter CDR3 length and a significantly larger size compared with “private” clonotypes. Taken together, shorter CDR3s highly enriched during thymic selection and antigen-driven selection, and further enriched in public T-cell responses. These results indicated that it may be evolutionary pressures drive short CDR3s to recognize most of antigen in nature.

## Introduction

To ward off a wide variety of pathogens, the human adaptive immune system harbors a vast array of TCRs, collectively referred to as the TCR-repertoire. The genes that encode the two primary types of TCRs, αβ, and γδ. 95% of T cells in humans are αβ T cells (1). Architecturally, the TCR β-chain is comprised of a variable (termed TRBV), diversity (TRBD), joining (TRBJ), and constant region (TRBC). The potential TCRβ repertoire consists of over 50 TRBV genes, two TRBD genes, 13 TRBJ genes, and two TRBC genes. Three hypervariable complementary determining regions (CDR) (CDR1, CDR2, CDR3) have been found in the variable regions of β-chain. The CDR1 and CDR2 loops are germline encoded by the TRBV genes, which predominantly interact with the MHC (2). The CDR3 nucleic acid sequence is the most diverse region, as it is generated by recombination of multiple V, D, and J gene segments, and by random trimming and addition of non-template nucleotides at the junction sites (N-diversity mechanisms), which greatly increases diversity further. In course of T-cell maturation, TCR locus recombination events can produce non-functional TCRs with frameshifts or stop codons (3). In this case, the T cell tries to arrange the second allele, and if the successful (in-frame) TCR formation occurs, the T cell carries both functional and non-functional TCR sequences (2, 4). Nonfunctional TCR sequences do not translate into functional TCRB chains, and cannot, therefore, subject to functional selection (positive and negative selection). Therefore, nonfunctional TCRs can be used to study the pre-selection TCR repertoire (5–7).

In the case of developing T cells, the assembly of in-frame receptor genes is low frequency event. Positively selected CD4^+^CD8^+^ double-positive thymocytes then start relocating to the medulla and differentiate into T-helper CD4^+^ single positive (SP) thymocytes that have the ability to recognize peptides presented by MHC class II molecules, or T-cytotoxic CD8^+^ SP thymocytes that can interact with MHC class I molecules (8). Subsequently, T cells with excessive self reactivity are deleted in the thymus, a process called negative selection (9). Through the above process and selection, generating the post-selection naive repertoire. Typically, only one in a hundred thymocytes are thought to be granted access to the periphery. In the periphery, antigen exposure further narrows the repertoire over time leading to clonal expansion of antigen-specific populations. An encounter with a cognate peptide-MHC complex can induce naive T cells expressing the CD45RA isomer to begin to express CD45RO (10). There are a dynamic relationship between the naive and memory T cell repertoires, which involves continuous thymic output of new naive T cells, homeostatic maintenance of the peripheral repertoire, and recruitment of naive T cells to the memory pool through episodic and persistent antigenic stimulation (11). Moreover, upon antigen encounter, the response of both CD4^+^ and CD8^+^ cells is deemed to be essential for mounting an efficient immune response (12).

Here we used deep sequencing technologies to study human TCRβ CDR3 repertoires, and compared the characteristics of CD4^+^ naive, CD4^+^ memory, CD8^+^ naive, CD8^+^ memory T cells, including TCR diversity, CDR3 length distributions, usage frequency of TRBV segments, and public TCR repertoires. The present analysis provided a precise and robust analysis of the relationship between naive and memory (CD4^+^ and CD8^+^) T cell repertoires. Selective processes in the thymus and periphery may be key in shaping the T-cell repertoire, and the influence of selection is of concern in this study. Our goal is to produce comprehensive, unrestricted profiles of TCRβ CDR3 for the key subsets of T cells isolated from the blood of healthy individuals at sequence-level resolution.

## Materials and Methods

### Subjects

Six healthy blood donors, aged from 45 to 60, were recruited into this study. All recruited healthy donors provided written informed consent. The study was performed according to the tenets of the Declaration of Helsinki and was approved by the Ethics Committee of the First Affiliated Hospital, College of Medicine, Zhejiang University, China (Ref No 2015-313).

### T Cell Isolation and RNA Extraction

Fresh PBMC from an average of 20 mL heparinized peripheral blood were isolated over Ficoll gradient. Four lymphocyte subsets, CD4^+^CD45RA^+^, CD4^+^CD45RO^+^, CD8^+^CD45RA^+^, and CD8^+^CD45RO^+^ were isolated by FACSAria (BD Biosciences) (Figure S1) using the following antibodies (all from BD Biosciences): anti-CD4 PerCP-Cy5.5 (OKT4), anti-CD8 FITC (RPA-T8), anti-CD45RA APC (HI100), and anti-CD45RO PE (UCHL-1). All the sorted populations contained over 1.2 million cells and were >95% pure, as confirmed by FACS analysis. The percentage of the four cell subsets from each individual donor was presented in Table S1. RNA was immediately extracted from sorted cells using TRIzol Reagent (Invitrogen) according to the manufacturer's instructions. Isolated RNA was used on its entirety for cDNA synthesis using the High Capacity cDNA Reverse Transcription Kit (Applied Biosystems).

### Sequencing of TCRβ Repertoires and Bioinformatic Analyses

To generate the template library for Genome Analyzer, a multiplex-PCR system was designed to amplify rearranged TCRβ CDR3 regions from the whole cDNA sample using 32 forward primers, each specific to a functional TCR Vβ segment, and 13 reverse primers, each specific to a TCR Jβ segment. The details of the method of library construction were derived from earlier published work (13, 14). In addition, the primers sequence of TRB V/J were shown in Table S2. The qualified libraries will amplify on cBot to generate the cluster on the flowcell, and the amplified flow cell was pair-end sequenced using the Illumina HiSeq platform, with a read length of 100 as the most frequently used sequencing strategy. Subsequently, we filtered the raw data, including adapter contamination. Reads with an average quality score lower than 15 (based on the Illumina 0–41 quality system, the sequencing error rate is 3% when the quality score is 15) were removed, and a threshold for the proportion of N bases was set as < 5%. Moreover, ext, a few bases with low quality (lower than 10) were trimmed according to the earlier published work (13, 14). After filtering, pair-end (PE) read pairs were merged into a single contig sequence. MiTCR software was used to correct the sequencing errors and PCR amplification bias (15). In addition, algorithms to eliminate PCR and sequencing errors for the Illumina platform was executed according to the previous description (13, 16). Subsequently, reads were compared against the MiTCR program (developed by MiLaboratory; https://github.com/milaboratory/mitcr), yielding for each TCRB read V, D, and J gene usage, CDR3 length (i.e., number of nucleotides from the codon coding for the second conserved Cys in the V gene to the codon coding for the conserved phenylalanine in the J gene), and number of nucleotides inserted/deleted (Indel) in the Vβ-Dβ and Dβ-Jβ junctions. In addition, the frequency of expression of each distinct DNA sequence, amino acid sequence, and V-J combination was determined. All data generated or analyzed during this study were available from https://pan.baidu.com/s/1srRVpB4sfXXsNuDZ-xwpuw. The diversity of the TCR repertoire was assessed based on earlier published work (17). TCRβ CDR3 length distribution was analyzed by computing and comparing the proportions of each length category in each donor. The mixed effects two-way ANOVA calculated *P*-values for overall differences. Sharing TCR repertoires among cell subsets were quantified by calculating the number of overlap nucleotide (or amino acid) clonotypes between cell subset 1 and cell subset 2 in each given individual. Interindividual sharing TCR repertoires were quantified by calculating the number of common amino acid sequences (or nucleotide sequences) in each sharing category (The number of individuals in which a TCRβ clonotype was observe). Then calculated the proportions of each sharing category in each given individual.

### HLA Typing

HLA class I and II alleles were identified by SBT-sanger sequence. gDNA was extracted from patient granulocytes, and HLA class I and II genes were amplified by PCR. The details of the method of HLA typing were derived from earlier published work (18).

### Statistical Analysis

If not otherwise stated, data were presented as the mean ± SD values or as percentages (%), and statistical analyses were conducted using the unpaired *t*-test, two-way ANOVA test, Pearson test or Mann-Whitney U test where appropriate. A two-tailed *P* < 0.05 was considered significant. Statistical analyses were performed using SPSS20.

## Results

We used next generation sequencing technology to investigate the TCRβ CDR3 repertoires of different T cell subsets (CD4^+^CD45RA^+^, 4RA; CD4^+^CD45RO^+^, 4RO; CD8^+^CD45RA^+^, 8RA; and CD8^+^CD45RO^+^, 8RO) that had been purified from normal human peripheral blood samples. In total, we obtained an average of 6.68 million sequencing reads from each of 24 samples using the Illumina sequencing platform. Low-quality reads were filtered for quality using previously described criteria. On average, 0.13% (range, 0.07–0.19%) of reads were filtered out using this procedure. From these sequence reads, an average of 6.54 million CDR3 intervals were identified, which contained an average of 414105, 210778, 164866, and 58313 unique nucleotide sequences per sample for 4RA, 4RO, 8RA, and 8RO group, respectively, after filtering of the redundant identical sequences within each sample. A portion of each library was comprised by the out-of-frame clonotypes representing the non-functional TCR sequences formed during the recombination step. The percentage of such sequences was different for each sample, varying in most cases from 4.14 to 12.32% (mean value, 7.14%). A detailed description of reads and clones distribution was displayed in Table S3. In addition, the result of HLA typing was presented in Table S4.

### Memory Repertoire Was Less Diverse Than Those of Naive T Cell

**Figure 1:**
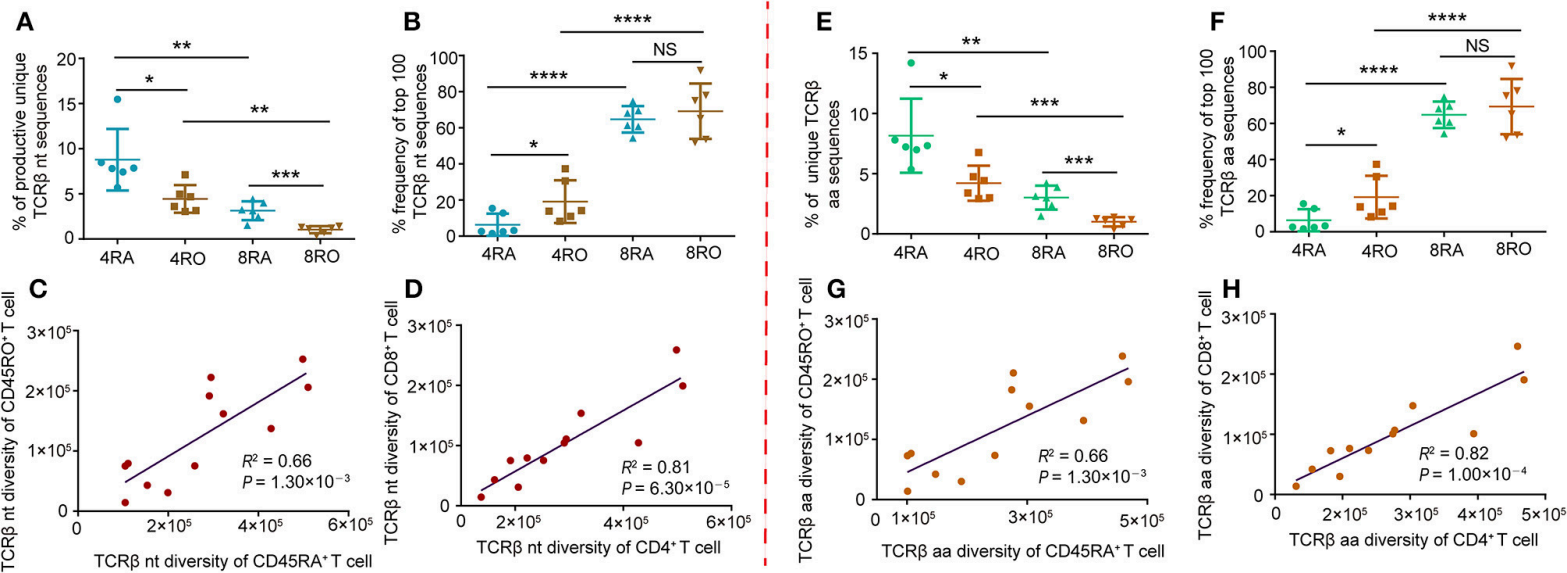
TCRβ CDR3 diversity analysis and correlation analysis of T-cell compartments in healthy donors. **(A)** Frequency of unique TCRβ nucleotide clonotypes identified in each sample of the different T-cell subsets. Data points represented the percentage of unique sequences in the total productive TCRβ repertoire of each individual. **(B)** Cumulative percentage frequency of top 100 TCRβ nucleotide clonotypes in each sample of the different T-cell subsets. Data points represented the cumulative percentage of the top 100 TCRβ nucleotide clonotypes in the total TCRβ repertoire of each sample. Data were presented as the mean ± SD values, and compared using the unpaired *t*-test. ^*^P < 0.05, ^**^P < 0.01, ^***^P < 0.001, ^****^P < 0.0001(two-tailed). **(C,D)** Sequencing data were normalized and true diversity indices positively correlate between CD45RA^+^ T cell subsets and CD45RO^+^ T cell subsets **(C)**, and positively correlate between CD4^+^ T cell subsets and CD8^+^ T cell subsets **(D)**, at the nucleotide level. **(E–H)** The same analysis was performed for amino acid clonotypes. nt, nucleotide; aa, amino acid; 4RA, CD4^+^CD45RA^+^ cells; 4RO, CD4^+^CD45RO^+^ cells; 8RA, CD8^+^CD45RA^+^ cells; 8RO, CD8^+^CD45RO^+^ cells.

Firstly, we characterized the entire TCRβ CDR3 profile of the CD4^+^/CD8^+^ naive and memory T-cell subsets (Figure S2). The frequency distribution showed the majority of the clonotypes was of low frequency in all the four T cell subsets, especially in naive CD4^+^ and CD8^+^ cells. High frequency clonotypes were increased in the memory CD4^+^ compartment, and even more so in the memory CD8^+^ cells. Subsequently, we investigated the TCRβ diversity of the four T-cell subsets using several evaluation methods. The percentage of unique clonotypes in the total TCRβ repertoire was calculated in each of the samples. This percentage was 8.79 ± 3.41%, 4.43 ± 1.53%, 3.14 ± 1.04%, and 1.03± 0.40% in the TCRβ nucleotide repertoires of 4RA, 4RO, 8RA, and 8RO group, respectively (Figure 1A). In addition, clonal expansion was further assessed by calculating the cumulative percentage of the repertoire that was constituted by the top 100 TCRβ nucleotide clonotypes (Figure 1B). The results showed that the rank of the diversity (from high to low) was 4RA, 4RO, 8RA, and 8RO. Interestingly, individuals with high diversity in the naive pool also have high diversity in the memory pool (Figure 1C), consistent with memory propagating from naive. Of note, this also applied to CD4^+^ pool and CD8^+^ pool, individuals with high diversity in the CD4^+^ pool also have high diversity in the CD8^+^ pool (Figure 1D). These differences in clonal sizes, TCRβ diversity, and correlations between each other at nucleotide level could underlie similar findings at amino acid level (Figures 1E–H). In addition, age may be a influence factor of repertoire diversity. However, in this study, we did not find any correlation between them (Figure S3).

### Shorter TCRβ CDR3s With Fewer Insertions Were Enriched During Thymic Selection

**Figure 2:**
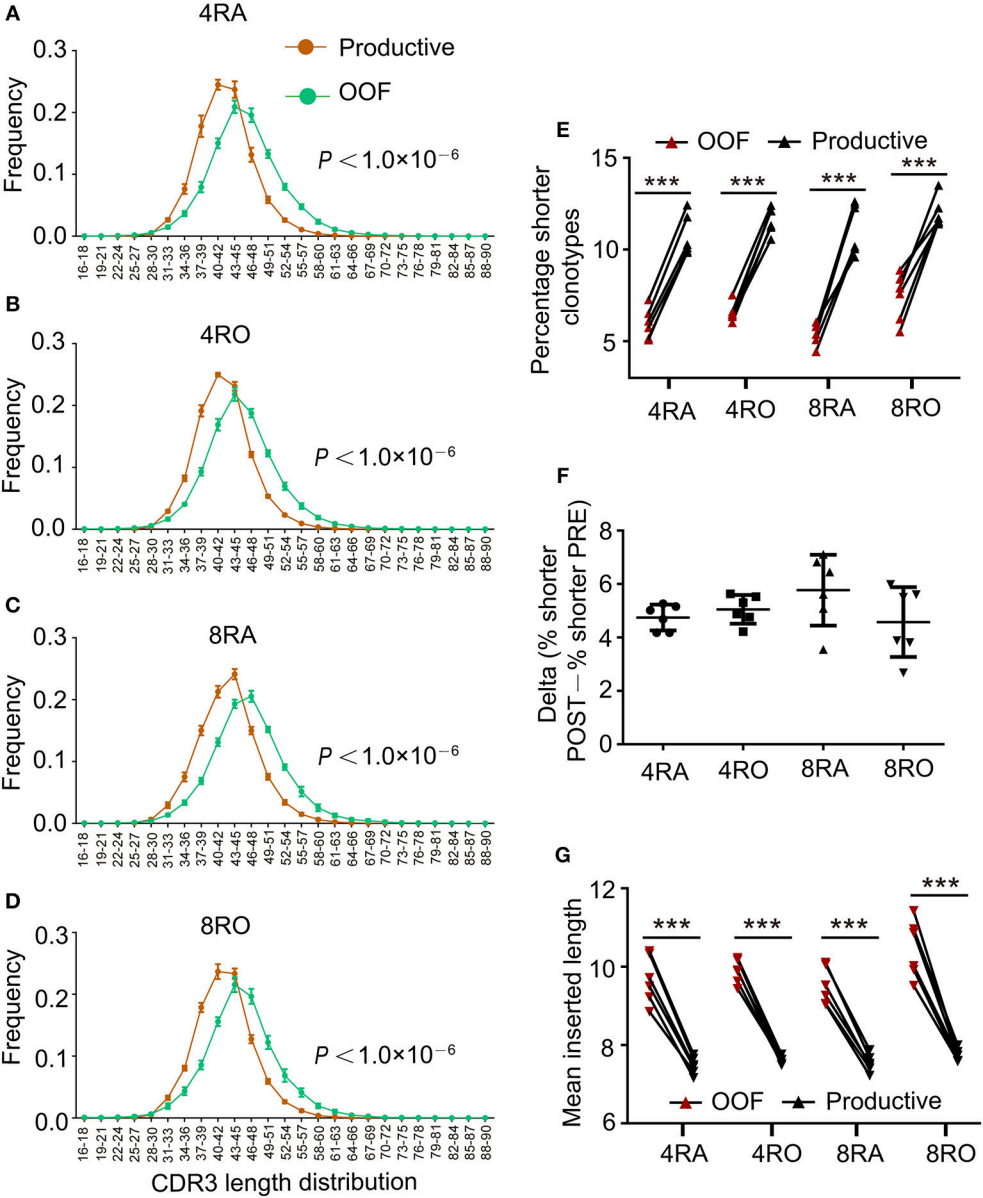
Shorter TCRβ CDR3s with fewer insertions were enriched during thymic selection. **(A–D)** Comparison of productive and out-of-frame TCRβ rearrangements revealed higher frequencies of short TCRβ CDR3s (and lower frequencies of long ones) in the Post-selection repertoires of 4RA **(A)**, 4RO **(B)**, 8RA **(C)**, and 8RO **(D)**. Mixed effects two-way ANOVA with individual as random variable. **(E)** The percentage of short TCRβ CDR3s clonotypes (16–36 nt) was obviously higher in the Post-selection repertoires compared with that in the Pre-selection repertoires in all the four T cell subsets. **(F)** There were no differences in the degree of enrichment in short TCRβ CDR3s among the four T cell subsets. **(G)** TCRβ CDR3s clonotypes with reduced insertions were enriched during thymic selection in all the four T cell subsets. Data were presented as the mean ± SD values, and compared using the unpaired *t*-test. ^***^P < 0.001(two-tailed).

TCRβ CDR3s repertoire might be affected by the selection in the thymus. Indeed, comparison of productive and out-of-frame TCRβ rearrangements in a pool of mixed cells revealed that high frequency of short CDR3s was observed in productive TCRβ sequences (Figures 2A–D), which indicating that shorter TCRβ CDR3s were enriched during thymic selection (Figure 2E), and a selection bias against long CDR3 loops. These findings were supported by the results from Gomez-Tourino et al (6). They also found that the percentage of shorter TCRβ CDR3s significantly increased after thymic selection. It was important to note, however, that four T cell subsets had similar degrees of enrichment of shorter clonotypes (Figure 2F). The mean inserted length was reduced significantly in Post-selection repertoires compared with Pre-selection repertoires (Figure 2G), reflecting a bias against long stretches of N nucleotide addition after selection (e.g., in 4RA, productive TCRβ CDR3 sequences contained on average 7.47 N additions at the V-D-J junction compared with 9.67 N additions in out-of-frame sequence, *p* < 0.001).

### Antigen-Experienced T Cells Harbor a Shorter CDR3

**Figure 3:**
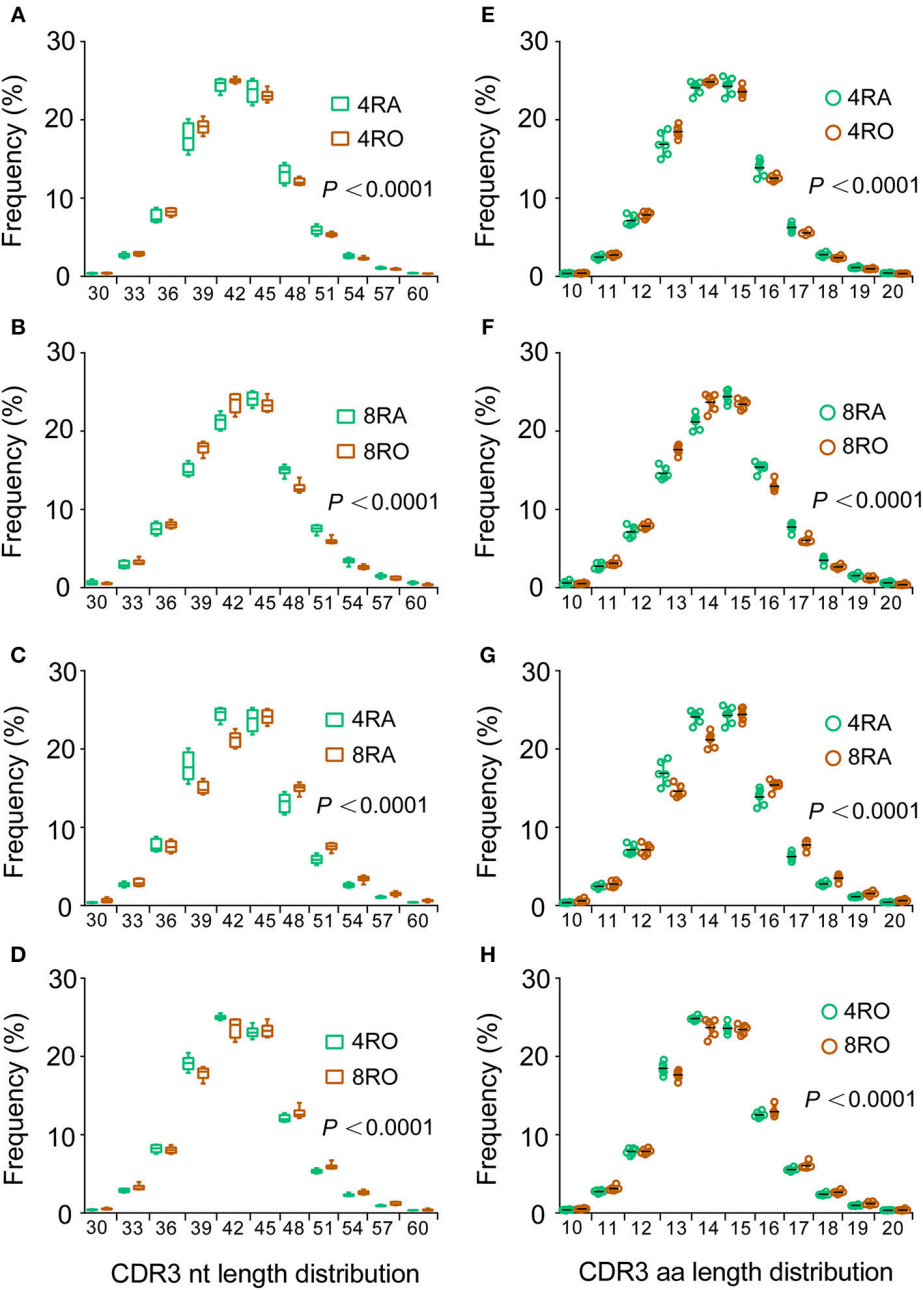
Memory T cells harbor shorter CDR3s vs. naive T cells and CD4+ T cells harbor shorter CDR3s vs. CD8+ T cells. **(A–D)** The length distributions of unique TCRβ CDR3 nucleotide sequences from CD4^+^ naive (4RA), CD4^+^ memory (4RO), CD8^+^ naive (4RA), and CD8^+^ memory (8RO) T cells were distinguished from one another. A significant reduction in CDR3 length was observed in memory T cells compared with naive T cells, no matter at the CD4+ cell level **(A)** or CD8+ cell level **(B)**; A significant reduction in CDR3 length was observed in CD4+ T cells compared with CD8+ T cells, no matter at the naive cell level **(C)** or memory cell level **(D)**, represented by higher frequencies of short TCRβ CDR3s (and lower frequencies of long ones) in memory T cells and CD4+ T cells (Mixed effects two-way ANOVA with individual as random variable). **(E–H)** The CDR3 length distributions of amino acid sequences displayed similar findings.

TCRβ CDR3 loops can vary in both length and sequence, allowing for the ability to recognize diverse antigens (19). The distribution of CDR3 sequence lengths is another feature that provides an overall view of repertoire composition. To avoid the distortion by dominant clones as results of immune responses, most of our following analyses (including CDR3 length distribution and VDJ gene usage) were carried out on unique nucleotide (or amino acid) sequences (irrespective of each clonotype frequency). We found that CDR3 length distributions differed between the naive and memory TCRβ repertoires, which showed a shift toward shorter clonotypes in memory cells vs. naive, no matter at the CD4+ cell level (Figure 3A) or CD8+ cell level (Figure 3B). In addition, a significant reduction in average CDR3 length was observed in CD4+ TCRβ repertoire compared with CD8+ TCRβ repertoire, no matter at the naive cell level (Figure 3C) or memory cell level (Figure 3D). Remarkably, and recapitulating our findings at the nucleotide level, the CDR3 length distributions of amino acid sequences displayed similar findings (Figures 3E–H). In order to verify the reliability of results, we filtered out all the rare clonotypes (clonotype abundance = 1) to reassess the CDR3 length distributions, due to the difficulty in distinguishing very rare sequences from sequencing errors. It is worth noticing that the results of robust test were consistent with the above findings (Figure S4).

### TRBV Segments Skewed in Short CDR3s Were Enriched in Memory Repertoire

**Figure 4:**
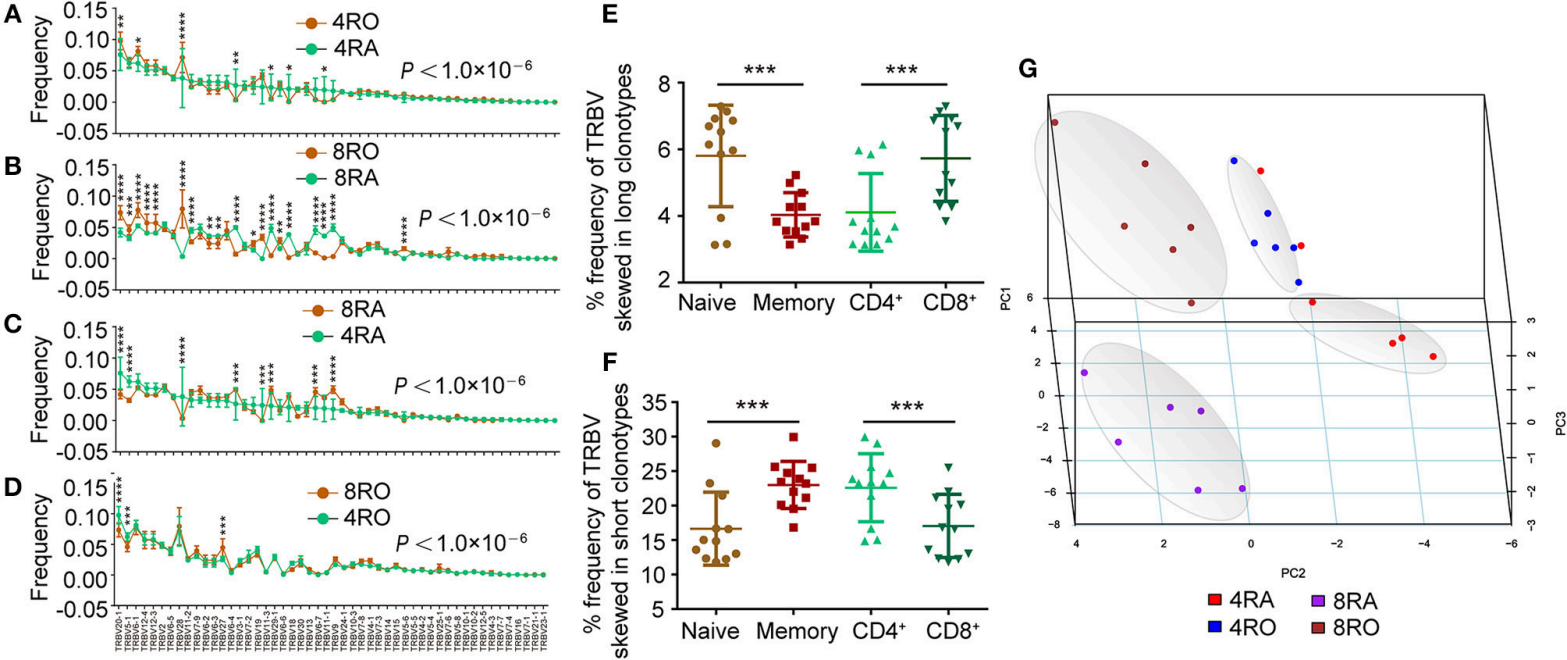
Usage frequency of TRBV genes segments are different between memory (CD8+) T cell and naive (CD4+) T cell. **(A–D)** Different usage frequency of TRBV genes between memory T cell and naive T cell were identified at CD4+ T cell level **(A)** and CD8+ T cell level **(B)**; Different usage frequency of TRBV genes between CD8+ T cell and CD4+ T cell were identified at naive T cell level **(C)** and memory T cell level **(D)**. The mixed effects two-way ANOVA calculated *P*-values for overall differences between T cell subsets. The single TRBV segment which was significant differences between T cell subsets was indicated by asterisk (Bonferroni correction). **(E,F)** Comparison of the frequency of TRBV segments which skewed in long CDR3s among the four T cell subsets **(E)**; Comparison of the frequency of TRBV segments which skewed in short CDR3s among the four T cell subsets **(F)**. **(G)** Principal coordinate analysis (PCA) based on TRBV segment usage showed profound differences among the four T cell subsets. Data were presented as the mean ± SD values, and compared using the unpaired *t*-test. ^*^P < 0.05, ^**^P < 0.01, ^***^P < 0.001, ^****^P < 0.0001 (two-tailed).

Moreover, we observed that usage frequency of TRBV segments was variable among the four T cell subsets (Figures 4A–D). TRBV20-1, TRBV28 showed higher usage, while TRBV6-4, TRBV11-3, TRBV6-6, and TRBV6-7 showed significantly lower usage in 4RO when compared with 4RA (Figure 4A). TRBV20-1, TRBV12-4, TRBV12-3, TRBV28, TRBV19, and TRBV15 showed higher usage, while TRBV11-2, TRBV6-2, TRBV6-3, TRBV6-4, TRBV11-3, TRBV6-6, TRBV13, TRBV6-7, and TRBV11-1 showed significantly lower usage in 8RO when compared with 8RA (Figure 4B). It was worth noticing that the specific skewed usages of TRBV20-1 and TRBV28 in memory T cell, TRBV6-4, TRBV11-3, TRBV6-6, and TRBV6-7 in naive T cell, could be observed in both CD4+ cell level and CD8^+^ cell level. Previous study had identified that some TRBV segments showed higher usage in long CDR3s, while some showed significantly higher usage in short CDR3s (13). Hereon, we found that the frequency of TRBV segments which skewed in long CDR3s was obviously higher in naive T cells and CD8^+^ T cells compared with that in memory T cells and CD4^+^ T cells, respectively (Figure 4E), and the frequency of TRBV segments which skewed in short CDR3s was obviously higher in memory T cells and CD4^+^ T cells (Figure 4F). From another point of view, this further proved previous discoveries that T cell subsets were distinguished in CDR3 lengths distribution. In addition, the four subsets of T cells could be distinguished from one another by principal coordinate analysis (PCA) based on TRBV segment usage (Figure 4G).

### Dominant Memory TCRβ Clonotypes Are Highly Represented in the Naive Pool

**Figure 5:**
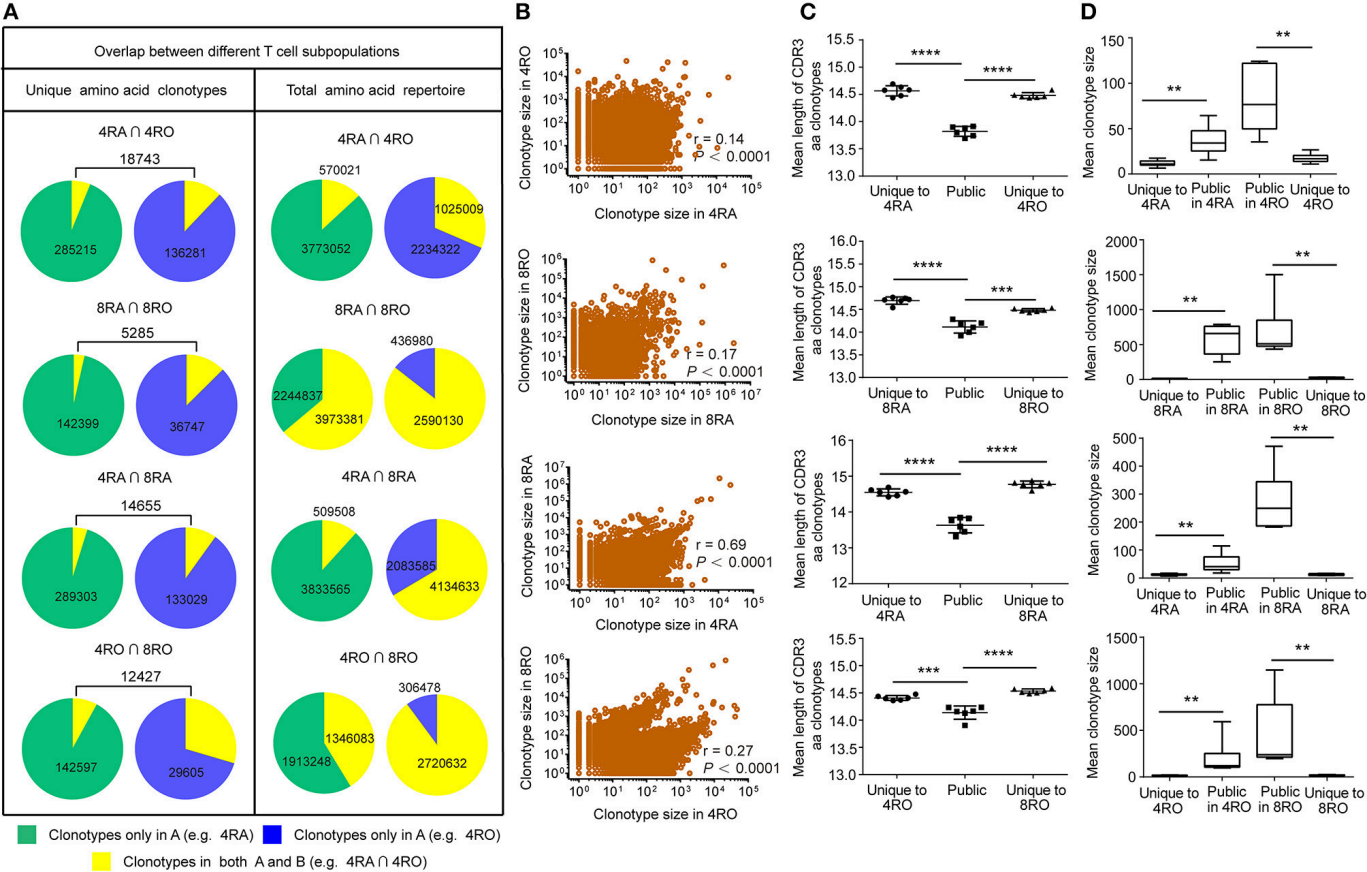
TCRβ amino acid clonotypes that were shared between naive and memory pools (CD4+ and CD8+ pools) in each donor. **(A)** The proportion of unique TCRβ amino acid clonotypes that were common to 4RA and 4RO pools, 8RA and 8RO pools, 4RA and 8RA pools, 4RO and 8RO pools in Donor 1 (Left panel). The proportion of the total TCRβ amino acid clonotypes that were common to 4RA and 4RO pools, 8RA and 8RO pools, 4RA and 8RA pools, 4RO and 8RO pools in Donor 1 (Right panel). **(B)** Scatter plot of relative abundance in the naive (CD4^+^) and memory (CD8^+^) compartments of all TCRβ amino acid clonotype that were observed in both compartments of the Donor 1. **(C,D)** TCRβ amino acid clonotypes common to the naive and memory pools (CD4+ and CD8+ pools) had a significantly shorter CDR3 length **(C)** and a significantly larger size **(D)** compared with those that were unique to one of the compartments. The statistics of CDR3 length and clonotype size were based on unpaired *t*-test and Mann-Whitney *U* test, respectively. ^**^P < 0.01, ^***^P < 0.001, ^****^P < 0.0001 (two-tailed).

TCRβ CDR3 clonotypes that are shared between cell subsets or between individuals are thought to play an important role in the efficacy of pathogen-specific responses and the control of infection. Therefore, we further examined the extent of overlap of TCRβ nucleotide clonotypes between the naive and memory T cell subsets, and between the CD4^+^ and CD8^+^ T cell subsets, at the nucleotide level and amino acid level (Figure 5A, and Figures S5–S8). In each donor, a part of TCRβ clonotypes were common to the memory (CD4^+^) and naive (CD8^+^) pools. of the unique TCRβ amino acid clonotypes in CD4^+^ (CD8^+^) memory pool of a donor, a mean of 13.77% (14.31%) was also present in the CD4^+^ (CD8^+^) naive pool. A much smaller percentage of the TCRβ amino acid clonotypes in the CD4^+^ (CD8^+^) naive repertoires was observed in the memory pools (CD4^+^: 7.01%, *P* < 0.01; CD8^+^: 5.10%, *P* < 0.001) Figures S5A, S6A, left panels). In addition, of the unique TCRβ nucleotide clonotypes in the CD8^+^ naive (memory) pool of a donor, a mean of 9.48% (27.78%) was also present in the CD4^+^ naive (memory) pool. A much smaller percentage of the TCRβ nucleotide clonotypes in the CD4^+^ naive (memory) repertoires was observed in the CD8+ pools (naive: 3.81%, *P* < 0.0001; memory: 7.97%, *P* < 0.001) Figures S7A, S8A, left panels). However, these findings provided limited indications of the dominant repertoire features within the overall repertoires. Therefore, we also assessed the degree of overlap between the naive (CD4^+^) and memory (CD8^+^) across the total TCRβ repertoires (including the size of each clonotype). Despite large interindividual variations, a substantial proportion of each donor's total memory (CD8^+^) TCRβ clonotypes were still highly represented in the naive (CD4^+^) pool, while a much smaller proportion of an individual's total naive (CD4^+^) TCRβ repertoire overlapped with the memory (CD8+) pool (Figures S5A–S8A, right panels). Remarkably, these differences of overlap degree at the amino acid level could underlie similar findings at the nucleotide level (Figures S5B–S8B). Furthermore, analyzing the part of TCRβ clonotypes that were observed in both the naive (CD4^+^) and memory (CD8^+^) subsets, we observed a positive correlation between the size with which individual clonotypes were observed in the naive (CD4^+^) compartment and their size in the memory (CD8^+^) compartment, no matter at the amino acid level (Figure 5B, and Figures S9A–S12A) or at the nucleotide level (Figures S9B-S12B).

### Public Sequences Is Characterized by Shorter CDR3 Length and Larger Size

**Figure 6:**
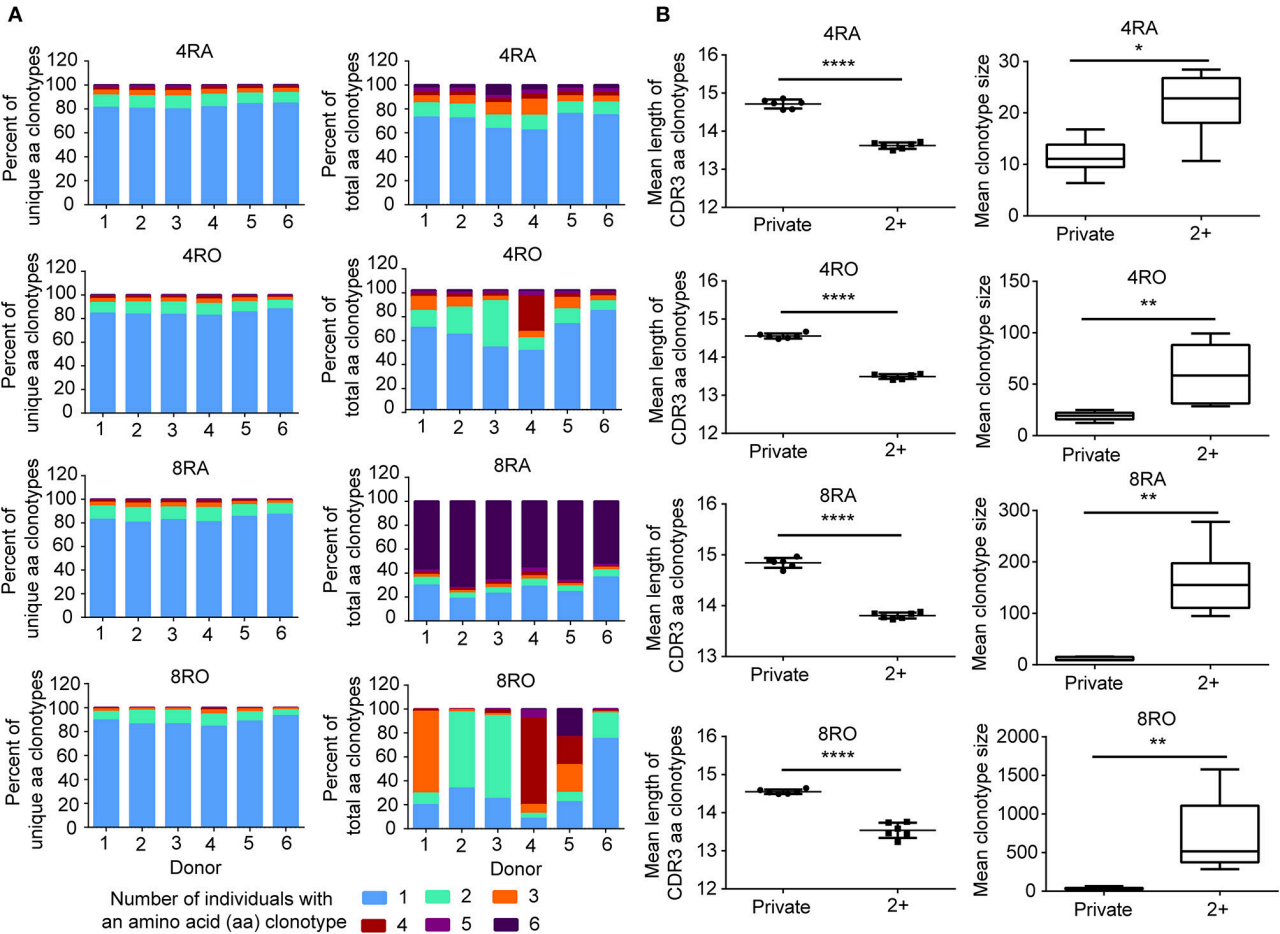
Interindividual sharing across all six donors of TCRβ amino acid clonotypes. **(A)** The number of individuals in which a TCRβ amino acid clonotype was observed, and the proportions of unique TCRβ amino acid clonotypes (Left panel) in the four T cell subsets repertoires of Donors 1–6 that were observed in one, two, three, four, five, or all six individuals. The same analysis was performed across the total TCRβ repertoires (including the size of each clonotype) (Right panel). **(B)** The TCRβ amino acid clonotypes which shared by ≥2 of 6 donors (2^+^) had a significantly shorter CDR3 length (Left panel) and a significantly larger size (Right panel) compared with those that presented only in a donor (Private), which observed in all the four T cell subsets (4RA, 4RO, 8RA, and 8RO). The statistics of CDR3 length and clonotype size were based on unpaired *t*-test and Mann-Whitney U test, respectively. ^*^P < 0.05, ^**^P < 0.01, ^****^P < 0.0001 (two-tailed).

To assess quantitatively the dominance of the TCRβ nucleotide clonotypes common to the naive and memory pools, or common to the CD4^+^ and CD8^+^ pools within an individual, we compared the size of these clonotypes with those that were unique to one of the subsets. At the amino acid level, we found that the TCRβ clonotypes common to naive (CD4^+^) and memory (CD8^+^) pools had a significantly shorter CDR3 length (Figure 5C) and a significantly larger size (Figure 5D) compared with those that were unique to one of the subsets. A similar tendencies could be observed at the nucleotide level (Figure S13). To further characterize private and public TCRβ CDR3 sequences, we did an analysis of interindividual sharing of identical TCRβ clonotypes, and found that there was a degree of TCRβ repertoire overlap between different individuals in each of the four T cell subsets. At the amino acid level, an average of 18.12, 15.55, 17.00, and 12.10% of the unique TCRβ clonotypes present, respectively in 4RA, 4RO, 8RA, and 8RO were shared by ≥2 of 6 donors (Figure 6A, left panels), which was significantly lower at the nucleotide level (4RA: 2.97%; 4RO: 2.79%; 8RA: 6.51%; 8RO: 4.84%) (Figure S14A, left panels). Second, as a measure of the interindividual overlap between total repertoires (including the size of each clonotype), we found that on average, 29.82, 35.56, 73.23, and 69.30% of their total TCRβ amino acid clonotypes in 4RA, 4RO, 8RA, and 8RO were shared by ≥2 of 6 donors (Figure 6A, right panels). It's still significantly lower at the nucleotide level (4RA: 10.30%; 4RO: 21.54%; 8RA: 68.94%; 8RO: 63.52%) (Figure S14A, right panels). Notably, the TCRβ clonotypes which shared by ≥2 of 6 donors had a significantly shorter CDR3 length and a significantly larger size compared with those that present only in one donor, and these findings could be observed in each of the four T cell subsets (4RA, 4RO, 8RA, and 8RO), no matter at the amino acid level (Figure 6B) or at the nucleotide level (Figure S14B). Human leukocyte antigen (HLA) types are likely to impact TCRβ features. However, in this study, we did not find that there was a association between the degree of overlap of HLA molecules and the public degree of TCRβ clonotypes (Figure S15).

## Discussion

An essential characteristic of T lymphocytes is their ability, as a population, to recognize an enormous number of peptide antigens. This capability is essential to the function of the adaptive immune system and is attributable to the diversity of the TCR they express. In the present study, we employed a more powerful deep-sequencing approach to analyze the TCRβ CDR3 repertoire of the four different T-cell subsets (4RA, 4RO, 8RA, and 8RO). We found that these four subsets of T cells are distinguished from one another in TCRβ diversity, CDR3 length distributions, usage frequency of TRBV segments, but a part of TCRβ clonotypes is common to these T cell subsets. The diversity of memory T cells is significantly lower than that of the naive T cells, at CD4^+^ cell level or CD8^+^ cell level. The diversity of CD4^+^ T cells is significantly greater than that of the CD8^+^ T cells, at naive T cell level or memory T cell level. The findings reported in the present study are in agreement with several previous studies (11, 20). The relative abundance of each specificity TCR is modulated by the individual's history of antigen exposure, as antigen-driven selection in the periphery leads to differential expansion of specific TCR clonotypes. It follows that TCR diversity is the highest in the naive compartment, with the antigen-experienced repertoire being skewed toward just some of these specificities.

**Figure 7:**
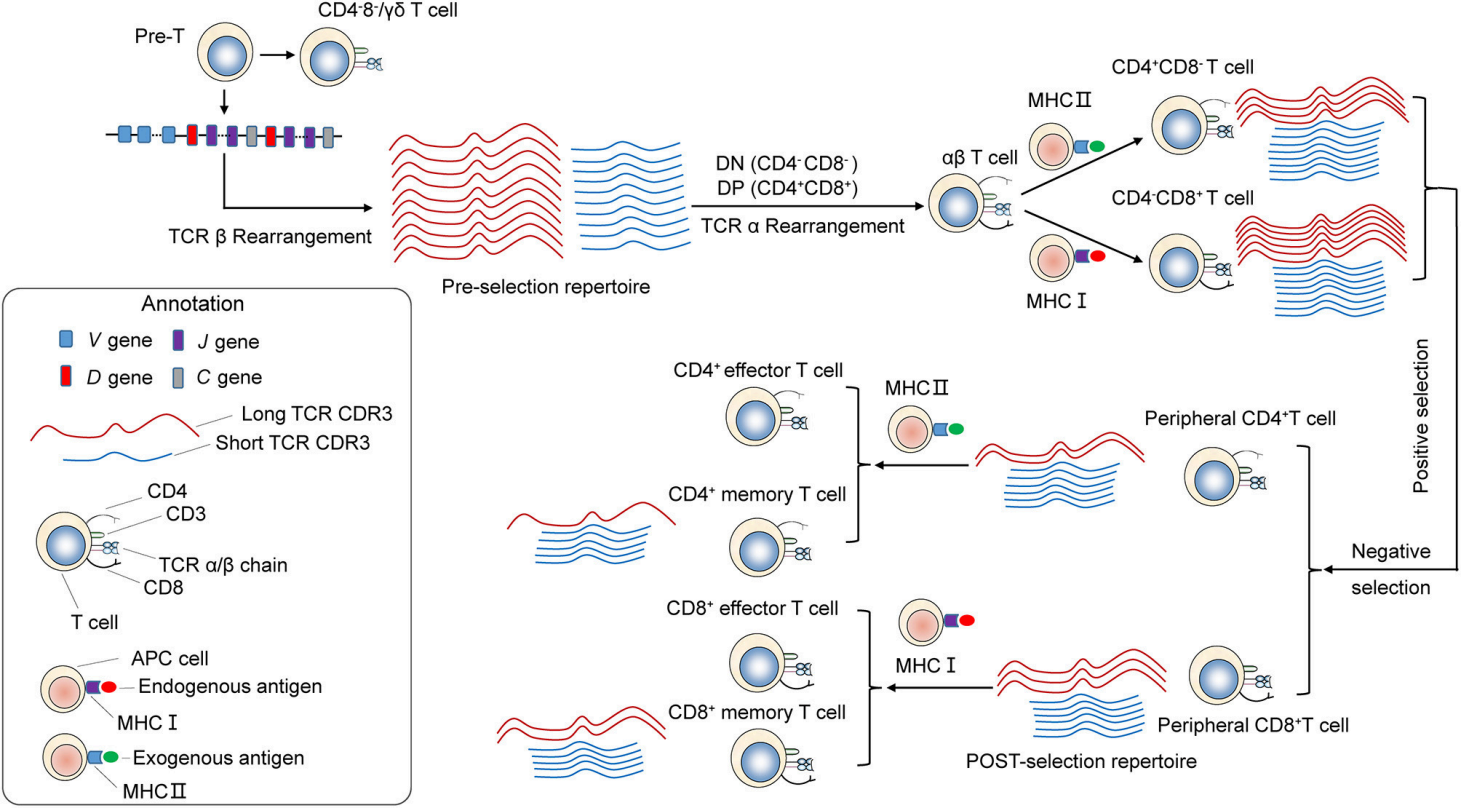
Skewing of the TCR repertoire during thymic selection and antigen-driven selection. T cells differentiate and mature through several thymocyte developmental stages. T-cell precursors differentiate into CD4^−^CD8^−^ [double-negative (DN)]. TCRβ gene locus rearrangement and expression first occur at the DN3 stage. A random combination of VDJ genes undergoes rearrangement, yielding a Pre-selection repertoire of short and long TCRB CDR3s. Subsequently, it becomes CD4^+^CD8^+^ double-positive (DP) cortical thymocytes. TCRα gene locus rearrangement take place in DP stage. As a result, functional TCRα/β heterodimer is expressed on the surface. These DP cells through a highly ordered developmental process called positive selection, during which engagement of TCR on DP cells by self-peptide MHC complex (pMHC) from thymic epithelial cells with appropriate avidity will deliver signals to DP cells and enable these cells to proceed into MHCII restricted CD4^+^ single positive or MHCI restricted CD8^+^ single positive thymocytes. Interaction with pMHCI may need longer TCRβ CDR3 than that of pMHCII. Therefore, a significant increase in average CDR3 size is observed in CD8^+^ T cells compared with CD4^+^ T cell. Then, T cells with excessive self reactivity are deleted in the thymus, a process called negative selection. Through the above process and selection, generating the Post-selection repertoire with an increased frequency of shorter TCRβ CDR3s, which undergo enrichment during thymic selection. The peripheral naive repertoire is shaped by antigen encounter. MHC Class I proteins are primarily involved with the presentation of intracellular antigen to cytotoxic CD8^+^ T cells, while MHC class II proteins present extracellular antigen to CD4^+^ T helper cells. It may be that short TCRβ CDR3s can be recognition of most antigen presented by MHC, and in result short CDR3s were further enriched in memory T cell repertoire.

The distribution of CDR3 sequence lengths is another feature that provides an overall view of repertoire composition. We found that shorter TCRβ CDR3s are enriched during thymic selection and antigen-driven selection. A model could be proposed to explain these findings (Figure 7). VDJ gene rearrangement typically yields a Pre-selection repertoire of TCRβ CDR3s across a range of lengths. Positive and negative selection in the thymus purges the pre-selection repertoire of most clonotypes. Most long CDR3 loops that do not survive the selection process. Short TCRβ CDR3 lengths, arise from reduced insertions during the TCRβ rearrangement process, were enriched. The CDR3 length distributions of CD4^+^ T cell is distinct from that of CD8^+^ cell, which may originate from the appropriate avidity of the engagement of TCR on double-positive (DP) cells by self-peptide MHC complex (pMHC) from thymic epithelial cells, which will deliver signals to DP cells and enable these cells to proceed into MHCII restricted CD4^+^ single positive (CD4^+^ SP) or MHCI restricted CD8^+^ SP thymocytes. Interaction with pMHCI may need longer TCRβ CDR3 than that of pMHCII. Therefore, a significant increase in average CDR3 length was observed in CD8^+^ T cells compared with CD4^+^ T cell. The net result of thymic selection is that the post-selection repertoire is largely purged of most clonotypes (21). Typically, only one in a hundred thymocytes are thought to be granted access to the periphery (9). The peripheral TCR repertoire is further shaped by antigen encounter and altered in the context of disease. Antigen-experienced T cells (memory T cells) have a shorter CDR3 than naive T cells. It may be that short TCRβ CDR3s can recognize most of antigen in the outside world, and in result short CDR3s are further enriched in memory T cell repertoire. The results from Afik et al. (22) had drawn similar conclusions, that the CDR3 sequence is significantly longer in YFV-specific CD8^+^ T cells with a naive-like state compared with those with an effector memory profile for both alpha and beta chains.

Public T-cell responses, in which T cells bearing identical T-cell receptors (TCRs) are observed to dominate the response to the same antigenic epitope in multiple individuals, have long been a focus of immune T-cell repertoire studies (1). A previous study by Li et al. (23) showed that there was little overlap in TCRβ sequences between CD4^+^ (0.3%) and CD8^+^ (1.3%) T cells. However, their analytical methods are different from ours. They firstly merged all the distinct CD4^+^/CD8^+^ TCRβ sequences in all of 8 individuals, then to analyze the overlap degree between CD4^+^ and CD8^+^ population. However, in our study, we analyze the overlap degree between CD4^+^ and CD8^+^ population in each donor (Figure 5). The overlap degree of the individual level should be larger than that of group level, because each individual has a different genetic background. In addition, we found that there is a high degree of overlap between the memory and naive repertoires within individuals. The results from Venturi et al. (11) had also drawn similar conclusions. It has been commonly thought that, following puberty, thymic function declines with age and that by 18 years of age the periphery is already seeded with a full complement of antigen-reactive T lymphocytes (24). However, many lines of evidence suggest that the adult thymus remains active late in life and contributes functional T cells (naive, newly differentiated T cells) to the peripheral lymphoid pool (25–27). Therefore, the presence of particular TCRβ clonotypes in the memory and naive pools evokes several interesting interpretations. one scenario is that naive T cells bearing the same TCR are entirely recruited into the memory pool during an antigen-specific response. The presence of those same TCRβ clonotypes in the naive pool would then derive from thymic replenishment of the naive repertoire with identical TCR sequences (11). Another interpretation is that asymmetric division and differentiation after antigen encounter result in T cells bearing the same TCRβ clonotypes having memory and naive phenotypes. Such a model has been proposed in mice (28). In conclusion, our results call attention to the substantial number of TCRβ clonotypes are shared between T cell subsets or between individuals. These public TCRβ clonotypes within subsets or interindividual tend to have shorter CDR3 length and a significantly larger size compared with “private” clonotypes, which may be a minimal level of repertoire diversity required for protection against the spectrum of commonly encountered pathogens (29, 30). Convergent recombination has been proposed as an explanation for the occurrence of “public” T-cell receptors (11, 31–33). It is hypothesized that public TCRs have a high probability of forming in the thymus. TCR sequences close to the germline (few insertions and deletions at the V-D and D-J junctions) appear to be created at a relatively high frequency. The repertoire features of public TCRs further proves our previous conclusion that shorter TCRβ CDR3s were enriched during thymic selection and antigen-driven selection. Based on these results, it may be explained from an evolutionary perspective. In order to adapt to the environmental factors, including disease-causing bacteria and viruses, immune system genes has been constantly improved. Although there are individual differences, human beings are exposed to a similar set of antigens processed in the same way for a long period of their life. This, in turn, suggests the possibility that the Vβ, Dβ, and Jβ segment sequences that contribute to recurrently generated TCRs could be subject to evolutionary pressures favoring sequences recognizing antigens from common pathogens. Generally, it is the survival of the fittest. Until today, shorts CDR3s can be recognition of most antigens in the nature. A recent studies have shown that mice deficient for Terminal deoxynucleotidyl transferase, the enzyme that catalyzes the template-independent insertion of nucleotides at the junctions, have 10-fold less diversity in their TCR CDR3 repertoires, with few insertions, yet these mice appear healthy, make efficient and specific immune responses, and display no increased susceptibility to infection (5).

In conclusion, we used high-throughput sequencing to study the TCRβ repertoires of CD4^+^ naive, CD4^+^ memory, CD8^+^ naive, and CD8^+^ memory. These analyses can provide information to distinguish different T-cell subsets, and gain a better understanding of the generation and evolution of TCRβ CDR3 repertoires in the adaptive immune system.

## Author Contributions

HD and XH conceived and designed the study. XH, PZ, and XZ performed the experiments and analyzed the data. XH, JC, and YL wrote the manuscript. JY, YY, and XL provided materials and technical support and contributed to helpful discussions and review of the final manuscript. All authors read and approved the final manuscript.

### Conflict of Interest Statement

The authors declare that the research was conducted in the absence of any commercial or financial relationships that could be construed as a potential conflict of interest. The reviewer LL declared a shared affiliation, though no other collaboration, with the authors to the handling editor.
