# Supplementary material for: Shorter TCR β-Chains Are Highly Enriched During Thymic Selection and Antigen-Driven Selection
Source: Front Immunol. 2019 Feb 26;10:299. doi: 10.3389/fimmu.2019.00299 (PMC6399399; doi:10.3389/fimmu.2019.00299)
Supplement: Supplementary file 1 [file Data_Sheet_1.PDF]

Supplementary Figures

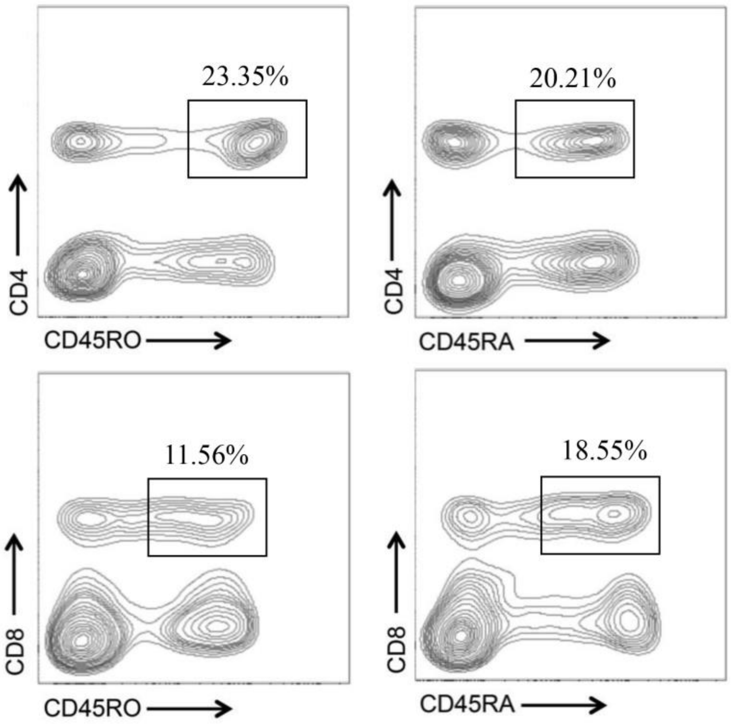

Figure S1. Isolation of CD4$^+$/CD8$^+$ naive and memory T cells by high-definition flow cytometric sorting. CD4$^+$ memory cells were defined as CD4$^+$CD8$^-$CD45RO$^+$CD45RA$^-$, and CD4+ naive cells were defined as CD4$^+$CD8$^-$CD45RO$^-$CD45RA$^+$. CD8$^+$ memory cells were categorized as CD4$^-$CD8$^+$CD45RO$^+$CD45RA$^-$, and CD8$^+$ naive cells were categorized as CD4$^-$CD8$^+$CD45RO$^-$CD45RA$^+$.

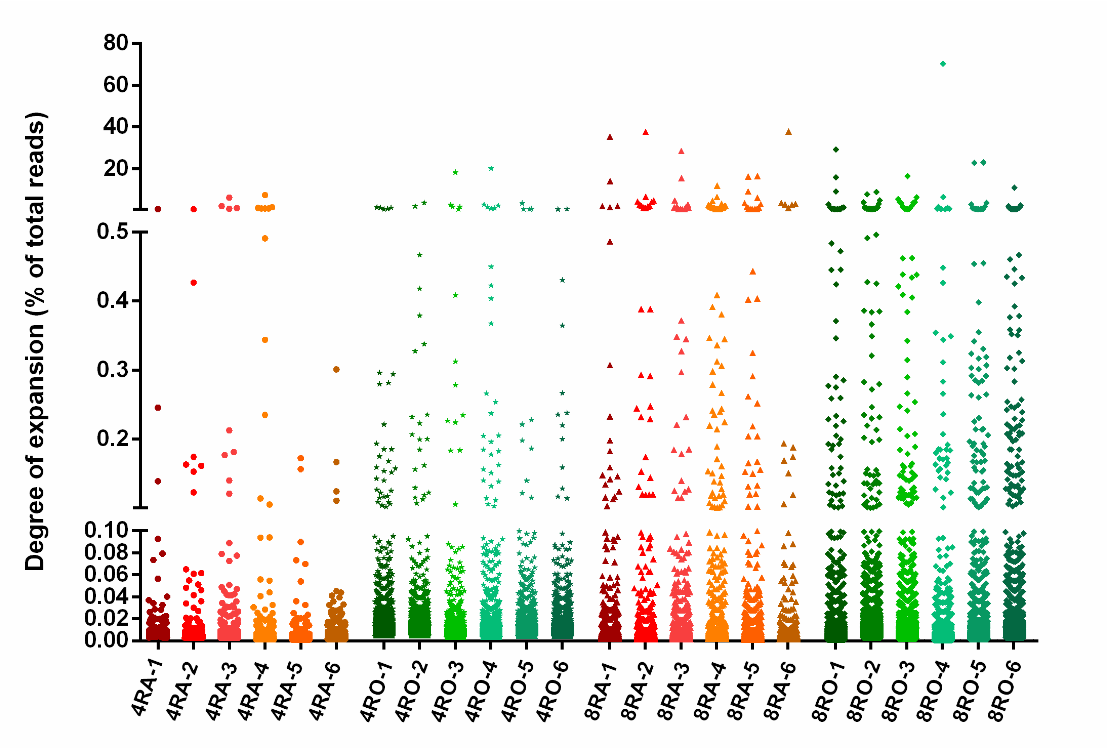

Figure S2. Degree of expansion and diversity analysis of TCRβ repertoire. Scatterplot showing all the unique nucleotide clonotypes that were recovered from each sample of the different T-cell subsets.

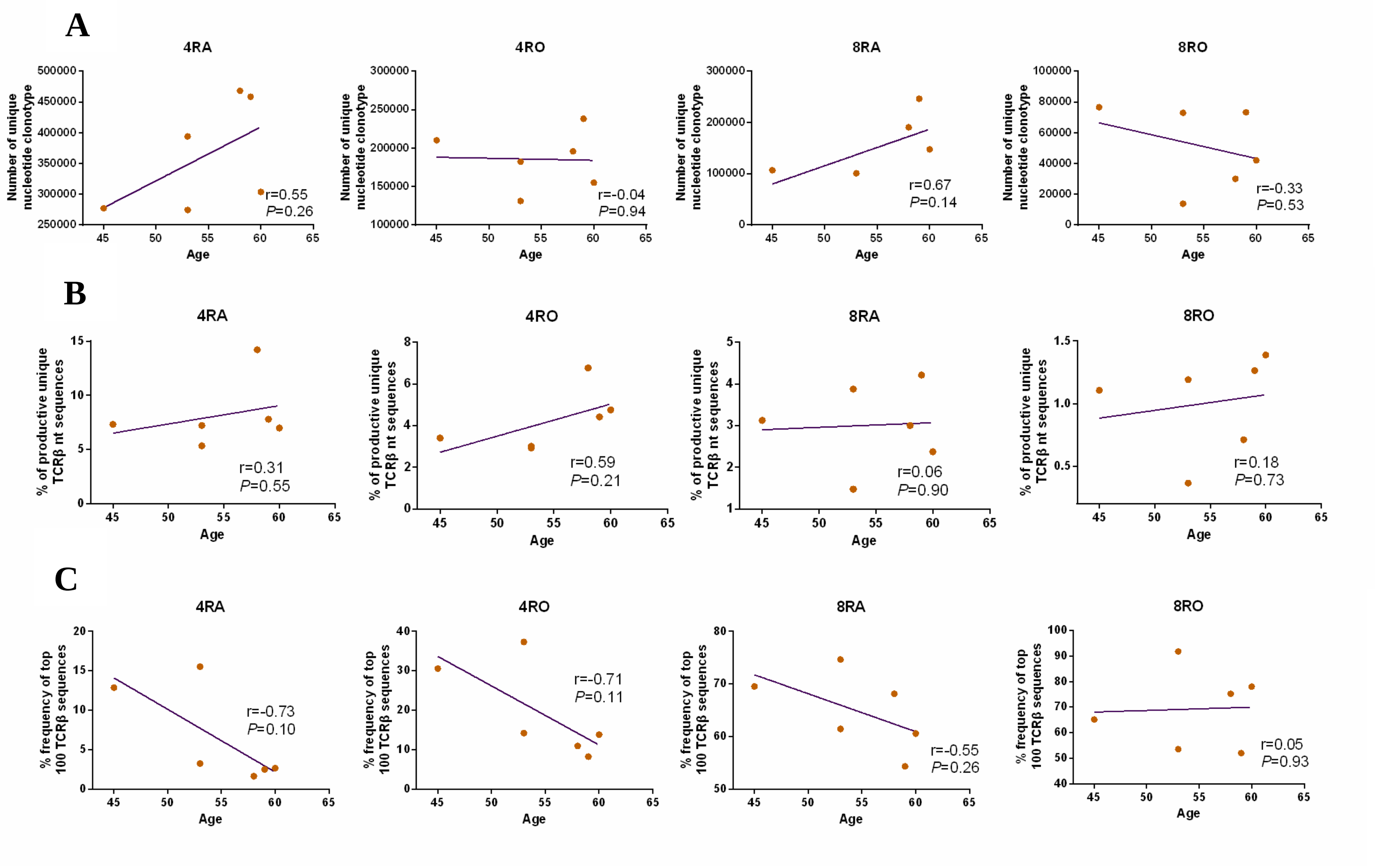

Figure S3. Correlation analysis between age and TCRβ CDR3 repertoire diversity. There was no correlation between age and TCRβ CDR3 repertoire diversity in all the four T cell subsets. Three diversity indicators were used for correlation analysis, including the true diversity indices (**A**), the percentage of unique amino acid clonotypes in the total TCRβ repertoire (**B**), and the cumulative percentage frequency of top 100 TCRβ amino acid clonotypes (**C**).

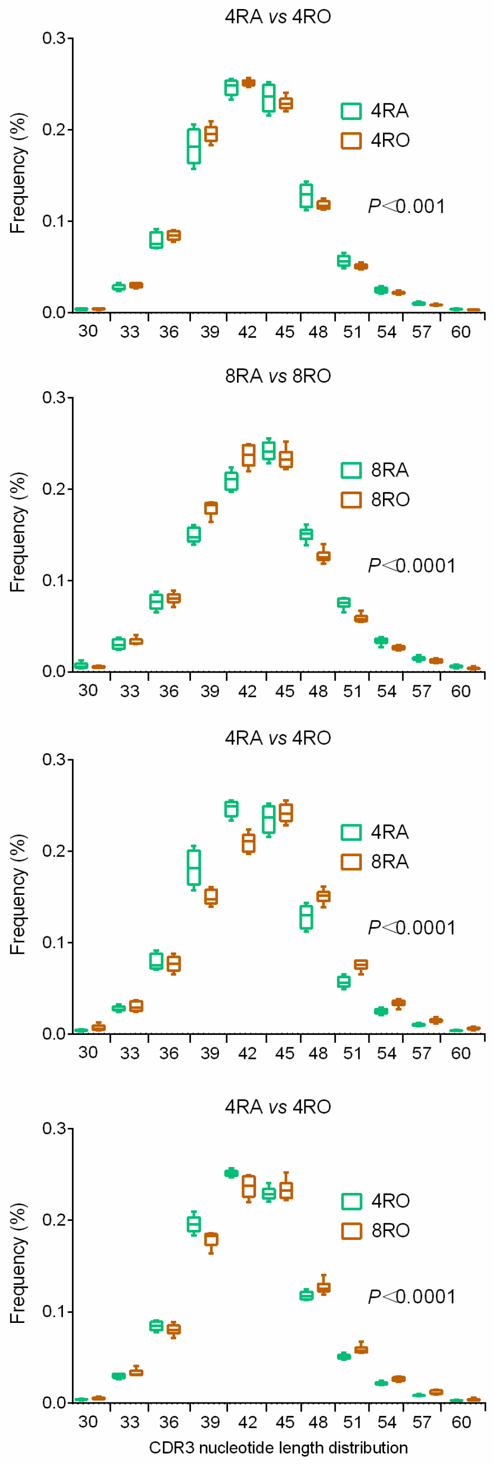

Figure S4. Re-estimate the CDR3 length distributions after filtering all rare clonotypes (clonotype abundance=1). To test whether the observed CDR3 length differences were an artificial error, all the rare clonotypes (clonotype abundance = 1) were filtered in each sample. TCRβ CDR3 nucleotide length distributions of $CD4^+$ naive (4RA), $CD4^+$ memory (4RO), $CD8^+$ naive (4RA) and $CD8^+$ memory (8RO) T cells were still distinguished from one another. A significant reduction in CDR3 length was observed in memory T cells and CD4+ T cells compared with naive and CD8+ T cells, respectively, represented by higher frequencies of short TCRβ CDR3s (and lower frequencies of long ones) in memory T cells and CD4+ T cells (Mixed effects two-way ANOVA with individual as random variable).

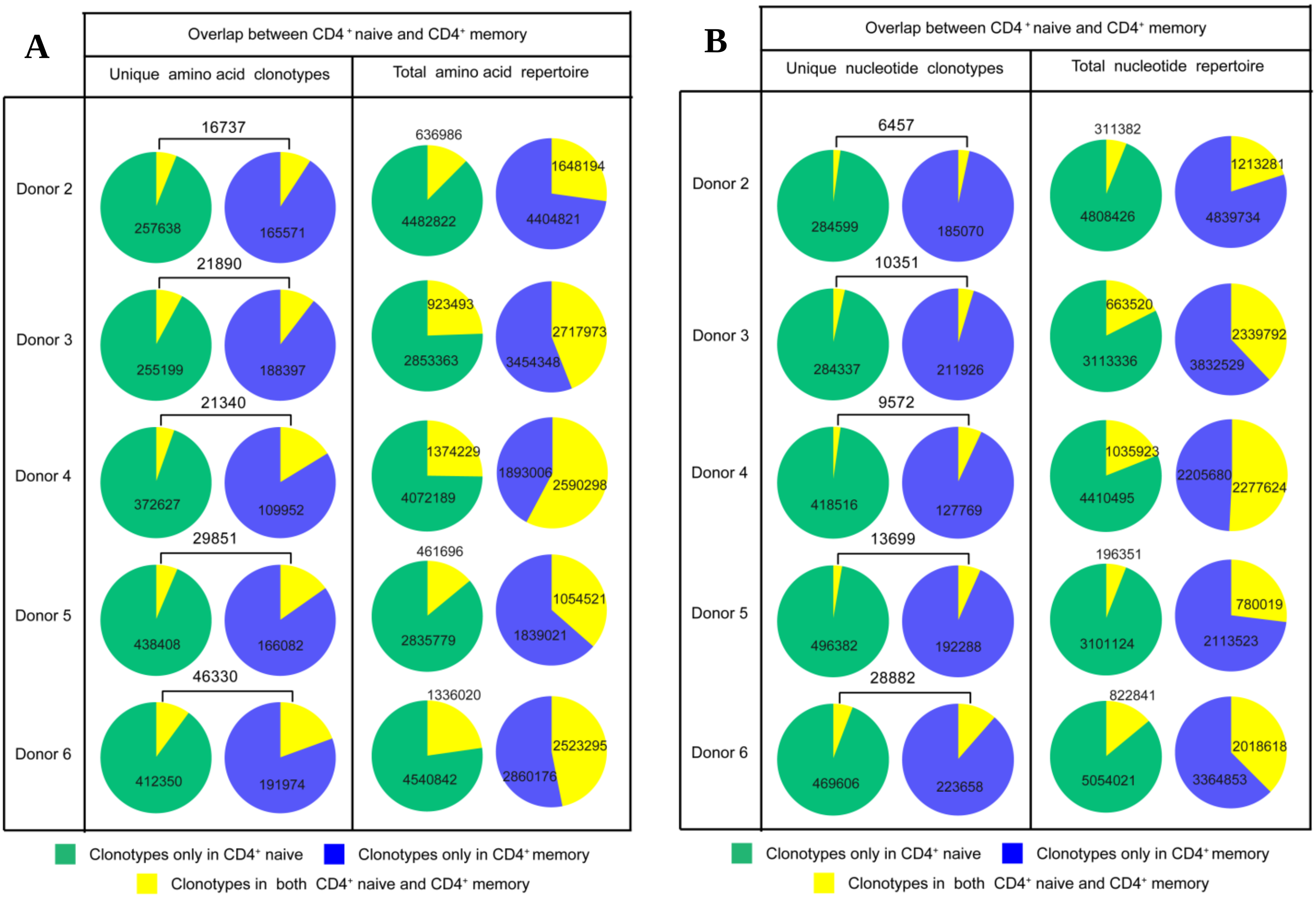

Figure S5. TCRβ clonotypes that were shared between $CD4^+$ naive and $CD4^+$ memory repertoires in each donor, at the amino acid level (**A**) and at the nucleotide level (**B**). Left panel, for the proportion of unique TCRβ clonotypes; Right panel, for the proportion of the total TCRβ repertoires.

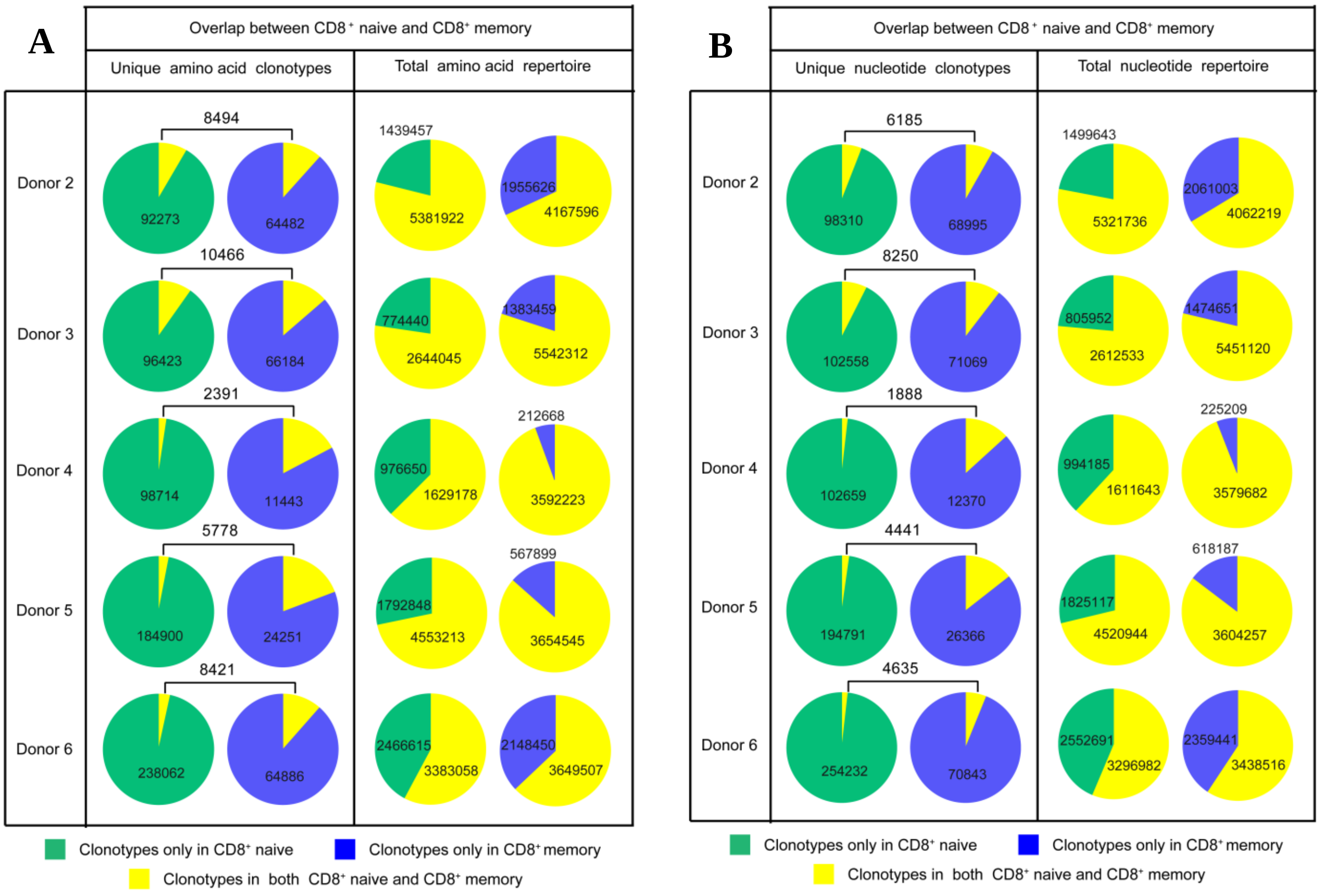

Figure S6. TCRβ clonotypes that were shared between CD8$^+$ naive and CD8$^+$ memory repertoires in each donor, at the amino acid level (**A**) and at the nucleotide level (**B**). Left panel, for the proportion of unique TCRβ clonotypes; Right panel, for the proportion of the total TCRβ repertoires.

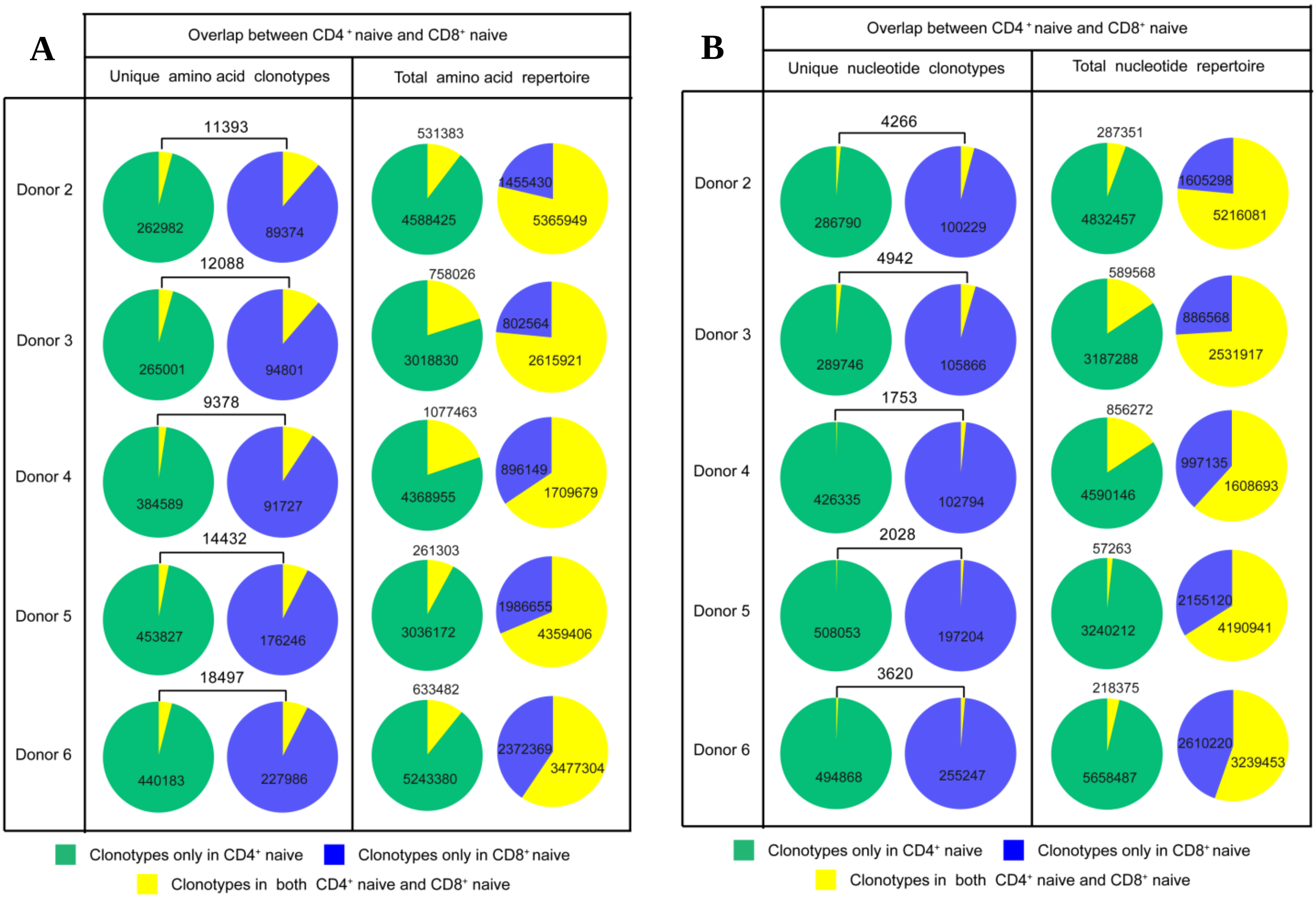

Figure S7. TCRβ clonotypes that were shared between CD4$^+$ naive and CD8$^+$ naive repertoires in each donor, at the amino acid level (**A**) and at the nucleotide level (**B**). Left panel, for the proportion of unique TCRβ clonotypes; Right panel, for the proportion of the total TCRβ repertoires.

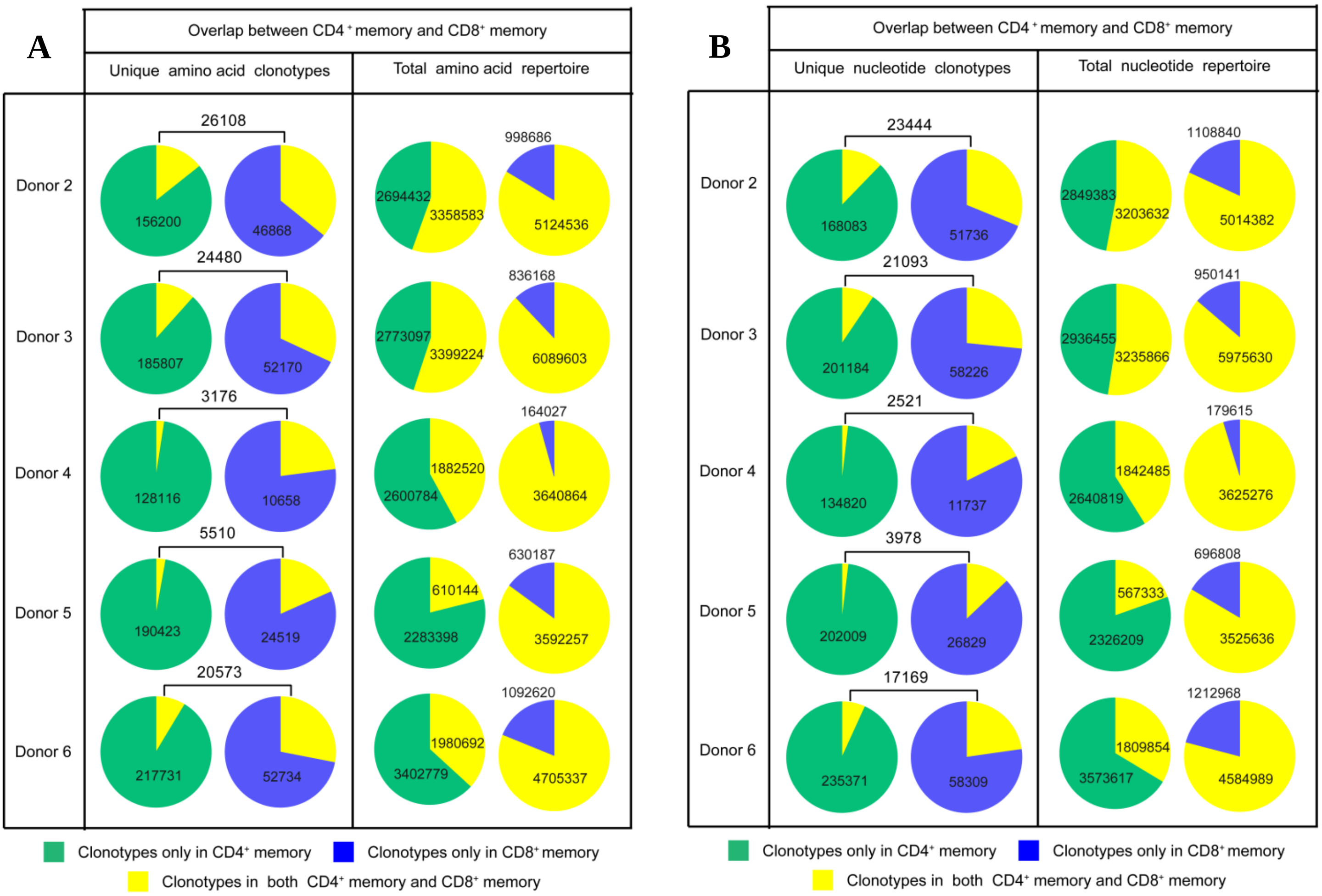

Figure S8. TCRβ clonotypes that were shared between CD4^{+} memory and CD8^{+} memory repertoires in each donor, at the amino acid level (**A**) and at the nucleotide level (**B**). Left panel, for the proportion of unique TCRβ clonotypes; Right panel, for the proportion of the total TCRβ repertoires.

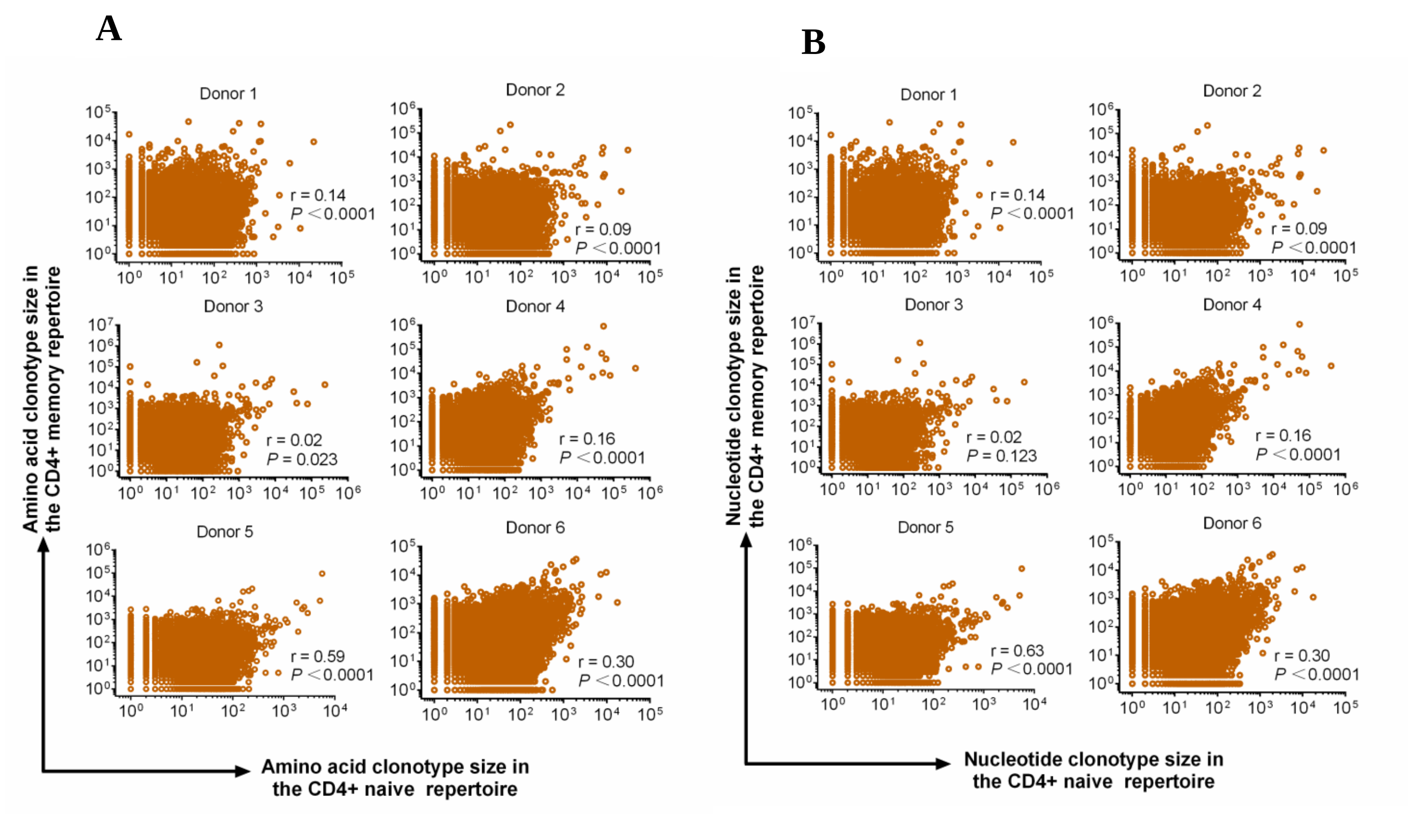

Figure S9. A positive correlation between TCRβ clonotype sizes in the $CD4^+$ naive and $CD4^+$ memory repertoires for clonotypes common to both pools in each donor, at the amino acid level (**A**) and at the nucleotide level (**B**). Correlations were based on the Spearman test.

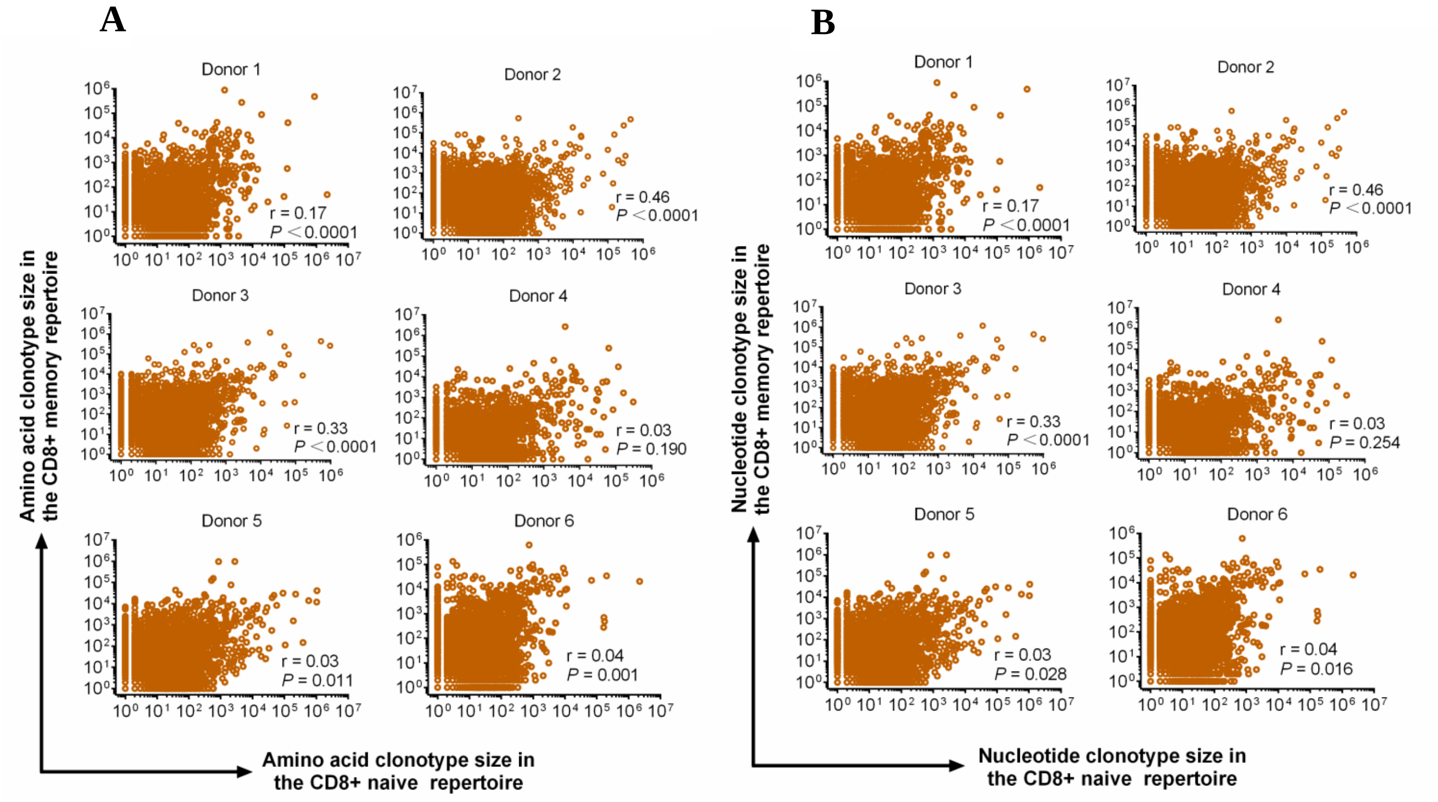

Figure S10. A positive correlation between TCRβ clonotype sizes in the $CD8^+$ naive and $CD8^+$ memory repertoires for clonotypes common to both pools in each donor, at the amino acid level (**A**) and at the nucleotide level (**B**). Correlations were based on the Spearman test.

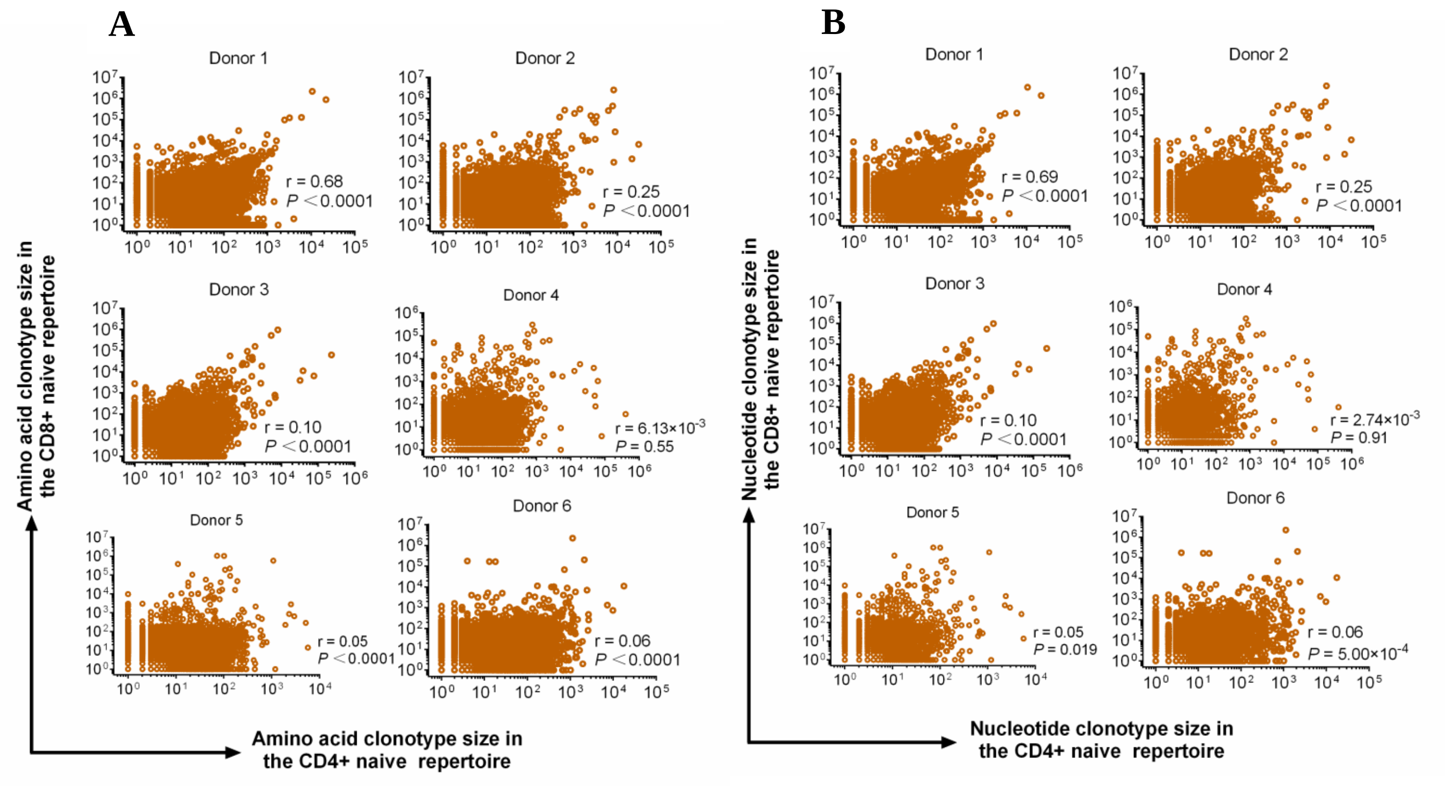

Figure S11. A positive correlation between TCRβ clonotype sizes in the CD4^{+} naive and CD8^{+} naive repertoires for clonotypes common to both pools in each donor, at the amino acid level (**A**) and at the nucleotide level (**B**). Correlations were based on the Spearman test.

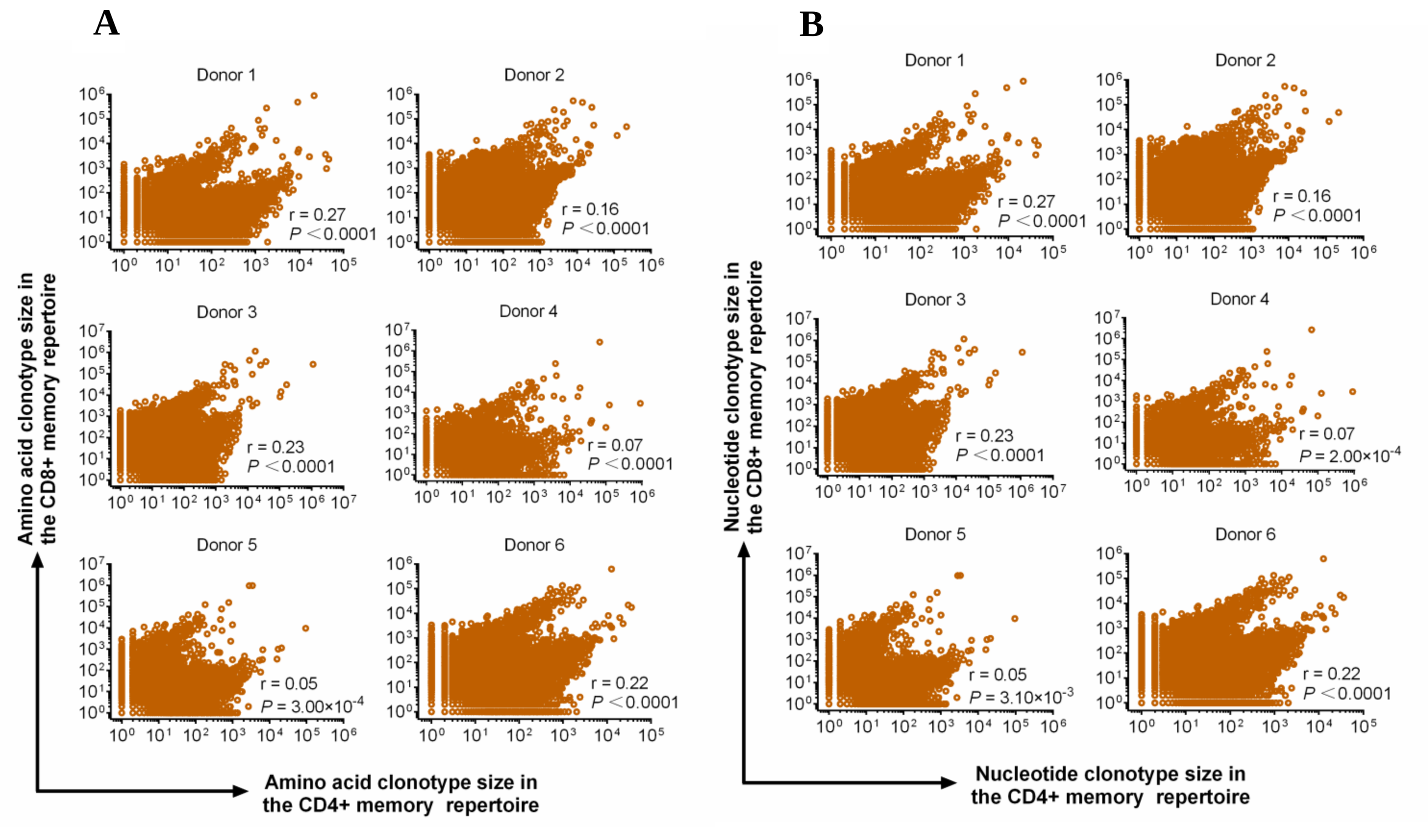

Figure S12. A positive correlation between TCRβ clonotype sizes in the $CD4^+$ memory and $CD8^+$ memory repertoires for clonotypes common to both pools in each donor, at the amino acid level (**A**) and at the nucleotide level (**B**). Correlations were based on the Spearman test.

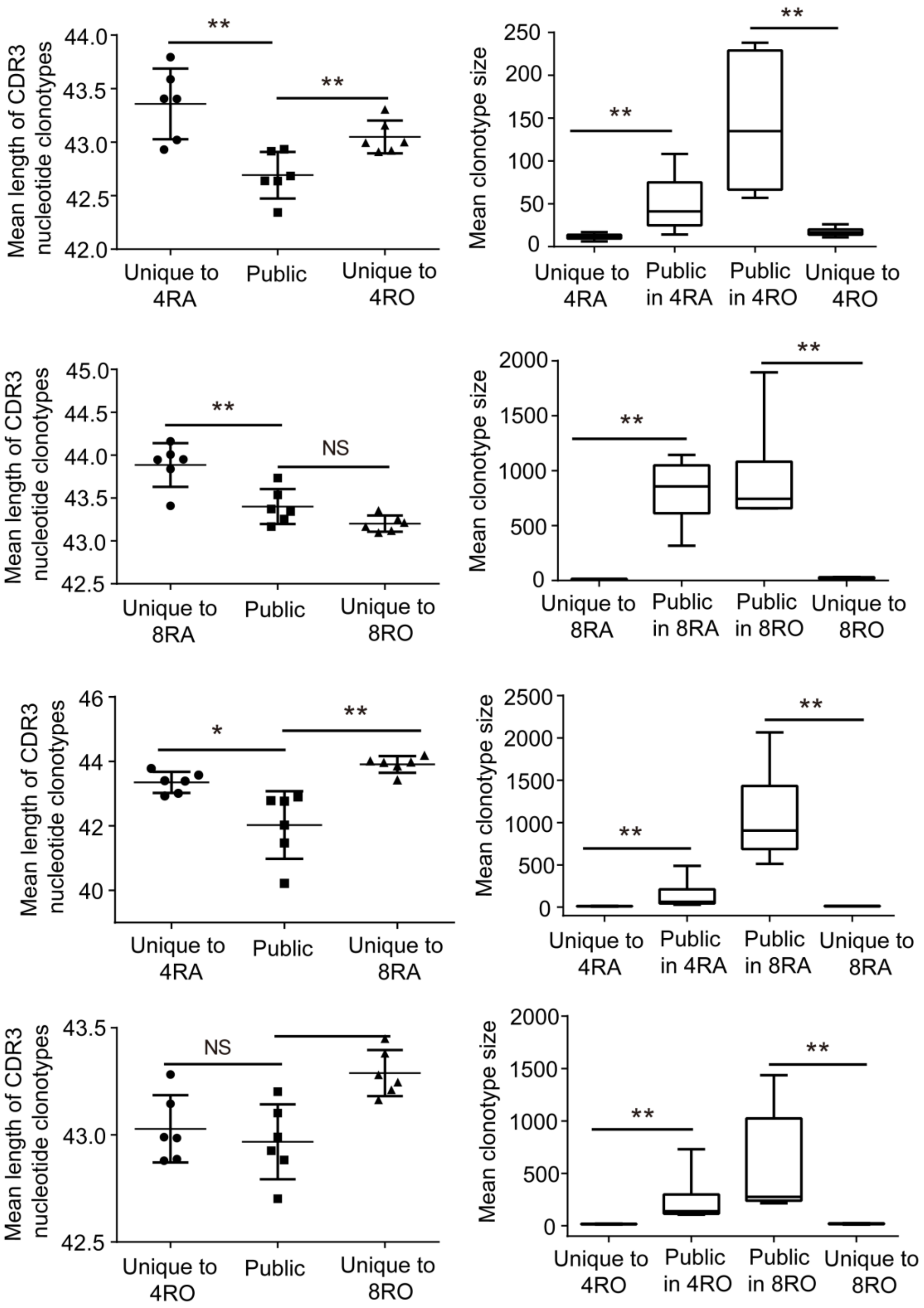

Figure S13. TCRβ nucleotide clonotypes common to the naive and memory pools ($CD4^+$ and $CD8^+$ pools) had a significantly shorter CDR3 length (Left panel) and a significantly larger size (Right panel) compared with those that were unique to one of the pools. The statistics of CDR3 length and clonotype size were based on unpaired t-test and Mann-Whitney U test, respectively. *$P < 0.05$, **$P < 0.01$, ***$P < 0.001$.

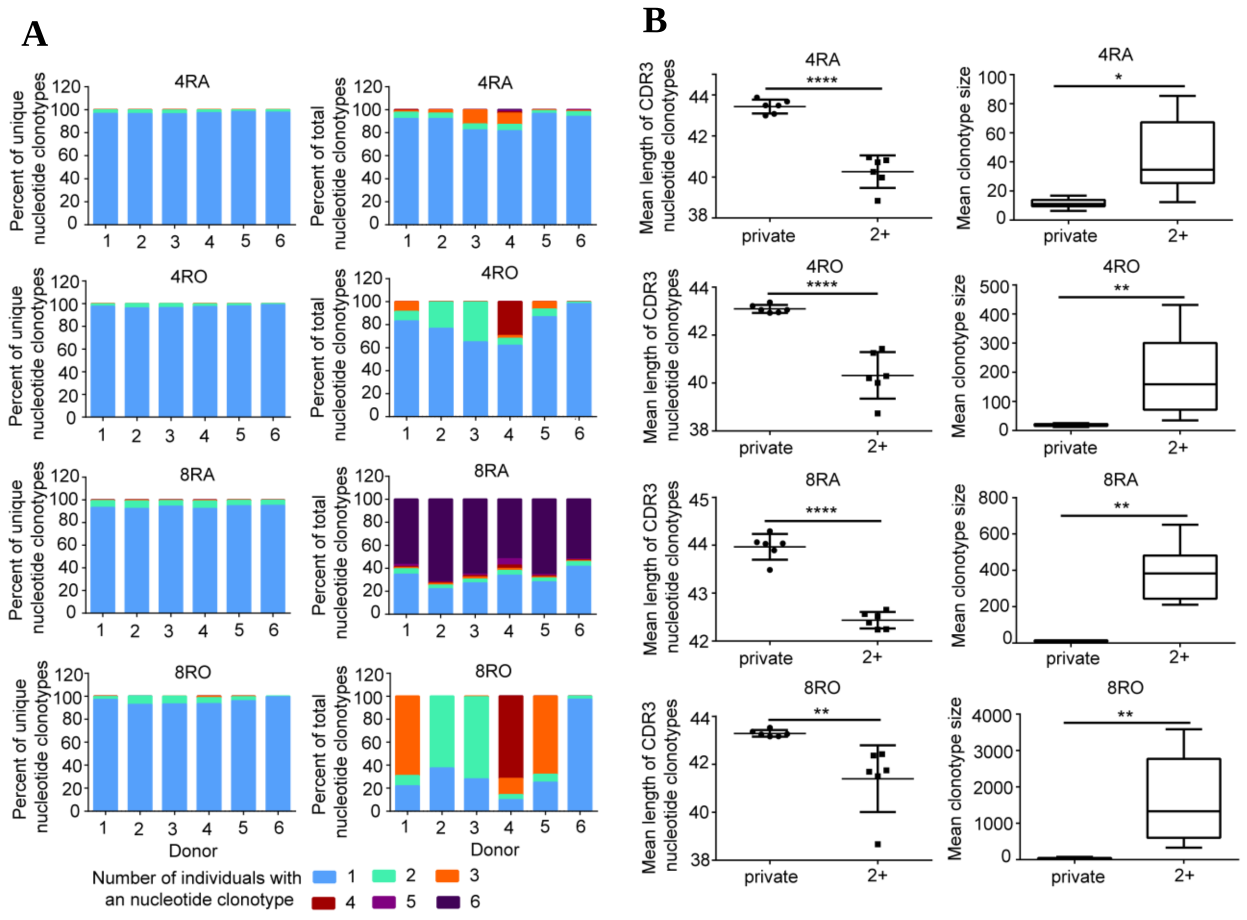

Figure S14. Interindividual sharing across all six donors of TCRβ nucleotide clonotypes. **A** The number of individuals in which a TCRβ nucleotide clonotype was observed, and the proportions of unique TCRβ nucleotide clonotypes (**Left panel**) in the four T cell subsets repertoires of Donors 1-6 that were observed in one, two, three, four, five, or all six individuals. The same analysis was performed across the total TCRβ repertoires (including the size of each clonotype) (**Right panel**). **B** The TCRβ nucleotide clonotypes which shared by ≥2 of 6 donors (2^{+}) had a significantly shorter CDR3 length (**Left panel**) and a significantly larger size (**Right panel**) compared with those that presented only in a donor (Private),which observed in all the four T cell subsets (4RA, 4RO, 8RA, and 8RO). The statistics of CDR3 length and clonotype size were based on unpaired t-test and Mann-Whitney U test, respectively. $*P < 0.05$, $**P < 0.01$, $***P < 0.001$, $****P < 0.0001$.

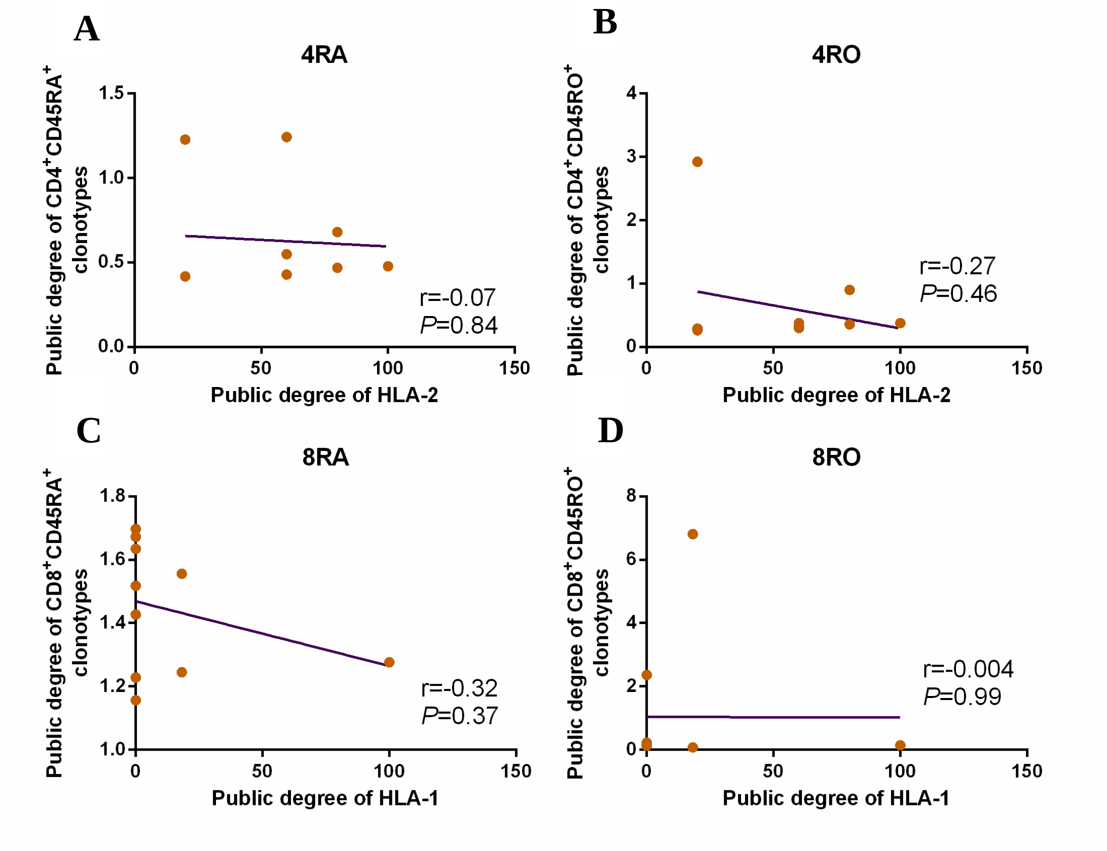

Figure S15. Correlations between HLA and public degree. **A, B** There was no correlation between the sharing of HLA class 2 alleles and the proportion of shared $CD4^+$ TCRβ nucleotide sequences, no matter at the naive cell level (**A**) or memory cell level (**B**). **C, D** There was no correlation between the sharing of HLA class 1 alleles and the proportion of shared $CD8^+$ TCRβ nucleotide sequences, no matter at the naive cell level (**C**) or memory cell level (**D**).

Supplementary Tables

Table S1. The percentage of the four cell subsets in each donor

Sample	$CD4^+$ naive T cell	$CD4^+$ memory T cell	$CD8^+$ naive T cell	$CD8^+$ memory T cell
HD1	19.57%	22.39%	17.52%	12.52%
HD2	18.72%	20.81%	18.80%	9.68%
HD3	21.31%	23.73%	17.83%	12.13%
HD4	20.79%	24.10%	19.08%	11.03%
HD5	19.10%	23.86%	18.16%	10.88%
HD6	21.74%	25.18%	19.89%	13.10%

Table S2. TRB V/J primers

TRB V Primers		TRB J Primers	
TRBV2	ATTTCACTCTGAAGATCCGGTCCAC	TRBJ1.1	CTTACCTACAACTGTGAGTCTGGTG
TRBV3-1	AAACAGTTCCAAATCGMTTCTCAC	TRBJ1.2	CTTACCTACAACGGTTAACCTGGTC
TRBV4-1/2/3	CAAGTCGCTTCTCACCTGAATG	TRBJ1.3	CTTACCTACAACAGTGAGCCAACTT
TRBV5-1	GCCAGTTCTCTAACTCTCGCTCT	TRBJ1.4	AAGACAGAGAGCTGGGTTCCACT
TRBV5-4/5/6/8	TCAGGTCGCCAGTTCCCTAAYTAT	TRBJ1.5	CTTACCTAGGATGGAGAGTCGAGTC
TRBV6-4.1	CACGTTGGCGTCTGCTGTACCCT	TRBJ1.6	CATACCTGTCACAGTGAGCCTG
TRBV6-8/5/1.2	CAGGCTGGTGTCGGCTGCTCCCT	TRBJ2.1	CCTTCTTACCTAGCACGGTGA
TRBV6-9/7/1.1/6	CAGGCTGGAGTCAGCTGCTCCCT	TRBJ2.2	CTTACCCAGTACGGTCAGCCT
TRBV6-4.2	AGTCGCTTGCTGTACCCTCTCAG	TRBJ2.3	CCGCTTACCGAGCACTGTCAG
TRRBV6-2/3	GGGGTTGGAGTCGGCTGCTCCCT	TRBJ2.4	AGCACTGAGAGCCGGGTCC
TRBV7-2/4/6/7/8	GGGATCCGTCTCCACTCTGAMGAT	TRBJ2.5	CGAGCACCAGGAGCCGCGT
TRBV7-3	GGGATCCGTCTCTACTCTGAAGAT	TRBJ2.6	CTCGCCCAGCACGGTCAGCCT
TRBV7-9	GGGATCTTTCTCCACCTTGGAGAT	TRBJ2.7	CTTACCTGTGACCGTGAGCCTG
TRBV9	CCTGACTTGCACTCTGAACTAAACCT		
TRBV10-1	CCTCACTCTGGAGTCTGCTGCC		
TRBV10-2/3	CCTCACTCTGGAGTCMGCTACC		
TRBV11-1/2/3	GCAGAGAGGCTCAAAGGAGTAGACT		
TRBV12-3.2/5.2	GAAGGTGCAGCCTGCAGAACCCAG		
TRBV12-3.1/4/5.1	GAAGATCCAGCCCTCAGAACCCAG		
TRBV13	TCGATTCTCAGCTCAACAGTTC		
TRBV14	GGAGGGACGTATTCTACTCTGAAGG		
TRBV15	TTCTTGACATCCGCTCACCAGG		
TRBV16	CTGTAGCCTTGAGATCCAGGCTACGA		
TRBV18	TAGATGAGTCAGGAATGCCAAAG		

TRBV19	TCCTTTCCTCTCACTGTGACATCGG
TRBV20-1	AACCATGCAAGCCTGACCTT
TRBV24-1	CTCCCTGTCCCTAGAGTCTGCCAT
TRBV25-1	GCCCTCACATACCTCTCAGTACCTC
TRBV27-1	GATCCTGGAGTCGCCCAGC
TRBV28	ATTCTGGAGTCCGCCAGC
TRBV29-1	AACTCTGACTGTGAGCAACATGAG
TRBV30-F5	CAGATCAGCTCTGAGGTGCCCCA

Table S3. TCRβ CDR3 sequence statistics

Sample	Total Reads (pair)	Filter rate (%)	All reads number	Total input sequences	Total good sequences	unique CDR3 nucleotide sequences	Out of frame clones(%)
HD1-4RA	8781091	0.14	8768909	8632742	4487429	347615	7.31
HD2-4RA	9385995	0.12	9374287	9231362	5234419	307521	5.37
HD3-4RA	8297001	0.16	8283897	8116398	3876389	311737	5.47
HD4-4RA	5741439	0.19	5730349	5668463	5569711	453239	5.55
HD5-4RA	3602206	0.11	3598274	3551444	3412236	540826	5.68
HD6-4RA	6893352	0.18	6880663	6783193	6014041	523693	4.81
HD1-4RO	3485535	0.13	3481115	3441578	3334988	177303	8.64
HD2-4RO	6419361	0.09	6413659	6241485	6137509	205504	6.8
HD3-4RO	6514801	0.08	6509567	6354763	6263286	241190	7.84
HD4-4RO	4755744	0.14	4749295	4701368	4564113	149459	8.11
HD5-4RO	3133745	0.10	3130455	3082130	2961559	221442	6.98
HD6-4RO	6201636	0.18	6190352	6103496	5470184	269772	6.39
HD1-8RA	11278706	0.11	11266648	11125516	6305601	166472	7.74
HD2-8RA	9708137	0.12	9696537	9542565	6978850	110870	5.75
HD3-8RA	7086848	0.12	7078183	6979615	3452806	116397	4.8
HD4-8RA	8041968	0.11	8033325	7930482	2957723	111393	6.15
HD5-8RA	10219147	0.13	10205879	10063299	6623495	214022	6.91
HD6-8RA	7670119	0.10	7662500	7541040	5905559	270039	4.14
HD1-8RO	3209023	0.14	3204534	3178965	3099946	47019	8.52
HD2-8RO	6814820	0.08	6809205	6485884	6400398	82720	9.12
HD3-8RO	7505746	0.07	7500289	7076757	6995032	87012	8.84

HD4-8RO	3980530	0.13	3975392	3939525	3822543	15858	10.09
HD5-8RO	4471204	0.14	4464802	4415607	4299509	35135	12.32
HD6-8RO	7030187	0.19	7016865	6933352	6283900	82133	8.10

Table S4. HLA typing for TCRβ repertoire profiling subjects

Subject	A		B		C		DRB1		DRB4		DQB1	
HD1	ND	ND	ND	ND	ND	ND	ND	ND	ND	ND	ND	ND
HD2	A*11:01	A*29:01	B*07:05	B*13:01	C*03:04	C*15:05	DRB1*07:01	DRB1*15:01	DRB4*01:03	DRB4*01:03	DQB1*06:01	DQB1*02:02
HD3	A*02:06	A*11:01	B*15:18	B*40:06	C*08:01	C*08:01	DRB1*04:04	DRB1*09:01	DRB4*01:03	DRB4*01:03	DQB1*03:02	DQB1*03:03
HD4	A*02:06	A*11:01	B*15:18	B*40:06	C*08:01	C*08:01	DRB1*04:04	DRB1*09:01	DRB4*01:03	DRB4*01:03	DQB1*03:02	DQB1*03:03
HD5	A*02:01	A*31:01	B*51:01	B*67:01	C*07:02	C*14:02	DRB1*04:03	DRB1*09:01	DRB4*01:03	DRB4*01:03	DQB1*03:02	DQB1*03:03
HD6	A*02:07	A*33:03	B*15:11	B*46:01	C*01:02	C*03:03	DRB1*09:01	DRB1*12:01	DRB4*01:03	DRB4*01:03	DQB1*03:01	DQB1*03:03

Abbreviation: ND, not detection.
